# Coexistence and cooperation in structured habitats

**DOI:** 10.1186/s12898-020-00281-y

**Published:** 2020-03-02

**Authors:** Lukas Geyrhofer, Naama Brenner

**Affiliations:** Network Biology Research Laboratories, and Department of Chemical Engineering, Technion–Israel Institute of Technology, Haifa, Israel

**Keywords:** Microbial interactions, Population dynamics, Multilevel selection, Public goods

## Abstract

**Background:**

Natural habitats are typically structured, imposing constraints on inhabiting populations and their interactions. Which conditions are important for coexistence of diverse communities, and how cooperative interaction stabilizes in such populations, have been important ecological and evolutionary questions.

**Results:**

We investigate a minimal ecological framework of microbial population dynamics that exhibits crucial features to show coexistence: Populations repeatedly undergo cycles of separation into compartmentalized habitats and mixing with new resources. The characteristic time-scale is longer than that typical of individual growth. Using analytic approximations, averaging techniques and phase-plane methods of dynamical systems, we provide a framework for analyzing various types of microbial interactions. Population composition and population size are both dynamic variables of the model; they are found to be decoupled both in terms of time-scale and parameter dependence. We present specific results for two examples of cooperative interaction by public goods: collective antibiotics resistance, and enhanced iron-availability by pyoverdine. We find stable coexistence to be a likely outcome.

**Conclusions:**

The two simple features of a long mixing time-scale and spatial compartmentalization are enough to enable coexisting strains. In particular, costly social traits are often stabilized in such an environment—and thus cooperation established.

## Background

The last few decades have seen an immense effort in trying to understand diverse communities of microbes [1–4]. They exist in biofilms, in guts of higher animals, and many other places, where they are important for ecological, economic, and medical affairs. Among the questions that have been asked are what is the origin of diversity in these communities, how can they survive and thrive together, and what role does a structured environment play? Empirical and theoretical answers point towards a few common themes. Endogenous mechanisms can support stable diversity of populations, for example by trade-offs in allocation between multiple resources [5, 6] and by mutualistic cross-feeding [7–9]. However, environmental factors may play a major role as well: Spatial structuring and compartmentalization are also found to contribute to diverse microbial populations and their mutual cooperation [10–19].

**Fig. 1:**
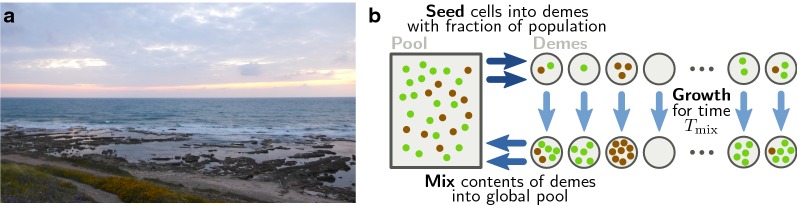
Spatio-temporally structured environments. **a** A rocky shore exposed to tidal cycles represents an example for structured environments considered in this article. Nutrients are replenished and contents of all small tidal pools are mixed during high tide, while allowing for segregated growth during low tide. The picture is taken by the authors and shows the coastline of Haifa (Israel) near Tel Shikmona. **b** Schematic depiction of cycles of growth, mixing and reseeding. Our model of many microbial populations growing in compartmentalized demes can be described by multilevel selection. Two levels are given by the growth dynamics within demes, and the cyclic dynamics of mixing and reseeding on a longer time-scale

A natural example of an ecological system with such strongly structured environments are tidal cycles on rocky shores [20–22] (see also Fig. 1): High tide dilutes populations into small tidal pools and replenishes nutrients, while at low tide remaining cells utilize these resources to replicate. The cyclic tidal dynamics may be more complex, but its crucial features include the spatial segregation of pools, and a new temporal scale determined by the tides, which is long compared to growth. Another scenario involving the same ingredients is the repeated colonization of surfaces and their dispersal [19]. Laboratory experiments can mimic these spatio-temporal ecological conditions, for instance by enclosing populations in milli- and micro-fluidic droplets [23–25], which are pooled and then seeded periodically into new droplets with fresh medium.

The ecological and environmental structuring of microbial communities allows to address also a more fundamental problem in evolutionary biology: The evolution of multicellular organisms from single celled ancestors. This required the formation of stable collectives engaged in cooperative interactions, providing further motivation to study the conditions for this to occur. Experimental studies showed that in yeast multicellular aggregates readily form when environmental conditions impose a selective advantage to groups of cells [26–28]. Different routes to multicellularity that do not involve cells staying together after dividing [29] exist as well: Slime molds come together and form multicellular bodies when resources are scarce [30]. Thus, environmental conditions can be seen as an *ecological scaffold* [31, 32], providing the necessary support for an major evolutionary transition [33, 34] of collectives into new individuals.

What are the minimal, or simplest, environmental conditions that support coexistence between competing microbial strains, allowing them to form stable and heterogeneous collectives? To what extent are these conditions sensitive to the details of the interaction, be it competitive or cooperative, between strains? To address these questions, we analyze a minimal model of environmental structuring, which combines spatial segregation with limited resources and a temporal cycle of mixing and reseeding. In the evolutionary literature, similar ingredients can be traced back to *trait group models* [35–39], that later led to the development of multilevel selection theory [40–42]. More recently, these concepts have been applied directly to microbial populations [10–13, 43]. Related concepts have also been developed in ecology with meta-community and meta-population approaches [44–46].

We develop a simple modeling framework to address interactions between microbial populations with different properties, including various implementations of interaction through public goods. Such a framework allows a direct comparison of specific biological interactions. In particular, we study growth on shared resources, the enzymatic degradation of antibiotic hazards, and resource extraction via siderophores. By analyzing the long-term dynamics of population composition and size, we show that stable coexistence between strains with different properties is a generic outcome in all cases. While the specific dynamical interactions are different, coexistence—and thus cooperation when public goods are involved—between strains is readily mediated by the spatio-temporal structuring of the environment.

## Methods and model

### Population dynamics in spatio-temporally structured environments

Our model describes several microbial populations growing in compartmentalized habitats (demes) that are repeatedly mixed. Within a deme they exhibit indirect interactions by competing for a single resource, and possibly also interact by producing public goods that affect this shared environment. After resources are depleted at time $T_\text{depl}$, growth terminates. At time $T_\text{mix}$ all demes are mixed into a common pool; this pool is diluted and cells are again seeded into empty demes with new resources. The cycle is repeated as illustrated schematically in Fig. 1b. We assume that $T_\text{mix}$ is slightly larger than $T_\text{depl}$ such that all resource is depleted, but additional processes like cell death are not yet an important aspect of the population dynamics.

The two phases of our dynamics—growth and mixing/dilution—are largely decoupled. We describe them by different mathematical tools (differential equations for growth, a stochastic mapping for mixing and dilution), and we use different notations to distinguish the relevant variables, which are summarized in Table 1.

**Table 1:** Notation used throughout the main text

Inoculum size	${\mathbf {n}}= (n_1,n_2,\ldots , n_i, \dots )$
Total inoculum size	$n = n_1 + n_2 + \cdots + n_i + \cdots$
Composition of inoculum	$x_i = n_i/n$
	$${\mathbf {x}}= (x_1,x_2,\ldots ,x_i,\ldots )$$
Seeding probabilities	${\mathbb {P}}\bigl [{\mathbf {n}} \bigl \vert \bigr .\, {\overline{n}},{\overline{{\mathbf {x}}}}\bigr ] = \prod _i\frac{({\overline{n}}\,{\overline{x}}_i)^{n_i}}{n_i!}e^{-{\overline{n}}\,{\overline{x}}_i}$
Averages over seeding	$\bigl \langle F(n,{\mathbf {x}}) \bigr \rangle = \sum _{{\mathbf {n}}}{\mathbb {P}}\bigl [{\mathbf {n}} \bigl \vert \bigr .\, {\overline{n}},{\overline{{\mathbf {x}}}}\bigr ]F(n,{\mathbf {x}})$
Cycle index	$(\tau )$
Mixing time	$T_\text{mix}$
Depletion time	$T_\text{depl}\bigl ({\mathbf {n}},\text{environment}\bigr )$
Within-deme observables	
Population sizes	${\mathbf {N}} = (N_1(t),N_2(t),\ldots )$
Population composition	${\mathbf {X}} = (X_1(t),X_2(t),\ldots )$
	$$X_i = N_i/N$$
Growth rate	$\alpha _i(t) = \alpha (1+\delta \alpha _i)A(t)$
Yield	$\varphi _i(t) = \varphi (1+\delta \varphi _i)Y(t)$
Resources	$S(t)$, $S(0) = S_0$
	$$\varphi \sim 1\Rightarrow N(T_\text{depl})\approx {\mathcal {O}}\bigl (S_0\bigr )$$
Depletion	$\alpha (t>T_\text{depl}) = 0$
Public good dynamics	
Production rates	$\varvec{\rho }= (\rho _1,\rho _2,\ldots ,\rho _i,\dots )$
	usually $\rho _1 > 0, \rho _i \approx 0, i\ge 2$
Antibiotics parameters	*B*(*t*); $$\kappa$$, $$\gamma$$, $$\mu$$$$\Rightarrow$$$$\alpha (t)$$
see “Collective reduction of antibiotics” section	
Pyoverdine parameters	*P*(*t*); $$\sigma$$$$\Rightarrow$$$$\varphi (t)$$
see “Iron extraction via siderophores” section	

### Dynamics within demes: the growth phase

A single deme is seeded with multiple strains, described by the *inoculum*${\mathbf {n}}= \bigl (n_1,n_2,\ldots \bigr )$, where $n_i$ is the number of cells of strain *i*. Within the growth phase, continuous time-dependent quantities are denoted by uppercase letters: These always include $N_i(t)$ for the population size of strain *i* and *S*(*t*) for resource concentration. In their general form the growth equations are

1a $$\begin{aligned} \dot{N}_i \,=\, & {} \alpha _iN_i\;, \end{aligned}$$

1b $$\begin{aligned} \dot{S}= & {} -\sum _i\frac{\alpha _iN_i}{\varphi _i}\;, \end{aligned}$$

where the dot denotes time derivatives, and strains are characterized by their growth rates $\alpha _i$ and yields $\varphi _i$. The sum over *i* in Eq. (1b) includes all strains, each consuming nutrients at their own rate. The inoculum size ${\mathbf {n}}$ provides the initial conditions for these dynamics, and the single resource is replenished to $S_0$ at the beginning of the growth phase. Many similar models where indirect interactions arise from shared nutrients are based on the MacArthur’s consumer-resource model [47, 48]. However, we will be interested in a more general scenario where indirect interactions are mediated through additional variables, not yet contained in the dynamics of population size and resource concentration, given by Eq. (1). These might be, for example, antibiotic concentration or additional resource dynamics, that lead to time-dependent growth rates $\alpha _i(t)$ or yields $\varphi _i(t)$. Note that in the model specified by Eq. (1), the finiteness of populations is implemented by the finiteness of resources; growth is stopped when resources are depleted. Technically, this is set by $\alpha _i(t)=0$ for $t>T_\text{depl}$. An alternative modeling approach that describes population finiteness is logistic growth. For our purposes including an explicit equation for the resource will be more convenient for describing the cooperative interactions, since it has the advantage that we can directly compare $T_\text{depl}$ to the mixing time $T_\text{mix}$.

In order to develop our approximations, a key assumption we make is to distinguish the differences between strains reflecting their intrinsic properties, from the effect of the environment on all strains. As long as cells are growing, we describe this situation as $\alpha _i(t) = \alpha (1+\delta \alpha _i)A(t)$, with $\alpha$ the average growth rate over all strains, $\delta \alpha _i$ are their relative differences that are assumed to be small ($\vert \delta \alpha _i\vert \ll 1$) and *A*(*t*) is a general time-dependent term that will depend on processes in the environment. Similarly, for yield we assume it is composed as $\varphi _i(t) = \varphi (1+\delta \varphi _i)Y(t)$, where $\varphi$ is the strain average, $\vert \delta \varphi _i\vert \ll 1$ are the relative differences between strains and *Y*(*t*) is the time-dependent coupling to the environment. While this approximation can be applied to many types of interactions, we will treat explicitly three specific cases: First, we establish a base behavior with both growth rate and yield constant in time, $A(t) = Y(t) = 1$. Then, we study collective antibiotic resistance that leads to time-varying growth rate, $A(t)\ne 1$, while the yield remains constant, $Y(t)=1$. Finally, pyoverdine production will be an example of time-dependent yield, *Y*(*t*) with a constant growth rate $A(t)=1$. With these definitions, $\alpha$ rescales time, and $\varphi$ defines the unit of substrate to generate one cell, such that both $\alpha t$ and $S\varphi$ are dimensionless numbers, describing time and the number of potentially growing cells, respectively.

### Dynamics among demes: cycles of mixing and reseeding

Since demes are seeded with the same replenished environment, the final population sizes only depend on the inoculum sizes ${\mathbf {n}}$. Thus, the growth phase can be represented in a coarse-grained form as a mapping between initial and final population size vectors

2 $$\begin{aligned} {\mathbf {n}}\mapsto {\mathbf {N}}(T_\text{mix};{\mathbf {n}})\;. \end{aligned}$$

After mixing contents of all demes into a common pool, it is diluted by a factor *d* and seeded into new demes. Thus, if the average population size of all demes in a previous cycle was $\bigl \langle {\mathbf {N}} \bigr \rangle$, the average at seeding will be $d\bigl \langle {\mathbf {N}} \bigr \rangle$. The seeded inoculum size ${\mathbf {n}}$ is assumed to follow a Poisson distribution ${\mathbb {P}}\bigl [{\mathbf {n}} \bigl \vert \bigr .\, d\bigl \langle {\mathbf {N}} \bigr \rangle \bigr ]$. This expression indicates the probability for each combination of ${\mathbf {n}}$, while the value after the vertical line denotes the average of this Poisson distribution. Therefore the re-seeding step is described by the mapping

3 $$\begin{aligned} \bigl \langle {\mathbf {N}} \bigr \rangle \mapsto {\mathbb {P}}\bigl [{\mathbf {n}} \bigl \vert \bigr .\, d\bigl \langle {\mathbf {N}} \bigr \rangle \bigr ]\;. \end{aligned}$$

The number of demes does not enter to the model—we only assume there are sufficiently many to describe the probability for seeding a certain combination with an independent Poisson distribution for each strain.

Combining growth with mixing and reseeding, the long-term dynamics takes the form of a mapping between cycles $\tau$ and $\tau +1$. Since the Poisson distribution is specified by its average, this mapping can be formulated between consecutive mean values ${\overline{{\mathbf {n}}}}^{(\tau )}$

4 $$\begin{aligned} {\overline{{\mathbf {n}}}}^{(\tau + 1)} =d \bigl \langle {\mathbf {N}} \bigr \rangle = d\sum \limits _{{\mathbf {n}}}{\mathbb {P}}\bigl [{\mathbf {n}} \bigl \vert \bigr .\, {\overline{{\mathbf {n}}}}^{(\tau )}\bigr ]{\mathbf {N}}(T_\text{mix};{\mathbf {n}})\;. \end{aligned}$$

Note the distinction in the two notations for averages: angular brackets $\bigl \langle \, \bigr \rangle$ indicate the *computation* of the average over all demes, as the weighted sum in Eq. (4). The overbar in ${\overline{{\mathbf {n}}}}^{(\tau )}$ indicates the *variable* for the average inoculum size, which changes over cycles. This second average acts as the parameter of the Poisson distribution for seeding.

Several important features of this mapping can be derived without specifying details of the growth phase. To derive these, we introduce total population sizes, $n = \sum _in_i$ at seeding and $N=\sum _iN_i$ at the end of growth phase; and fractions $x_i = n_i/n$ and $X_i=N_i/N$, denoted as vectors ${\mathbf {x}}$ and ${\mathbf {X}}$. It is straightforward to write the mapping in terms of differences $\Delta {\overline{n}}^{(\tau +1)} = {\overline{n}}^{(\tau +1)}-{\overline{n}}^{(\tau )}$ and $\Delta {\overline{{\mathbf {x}}}}^{(\tau +1)} = {\overline{{\mathbf {x}}}}^{(\tau +1)}-{\overline{{\mathbf {x}}}}^{(\tau )}$,

5a $$\begin{aligned} \Delta {\overline{n}}^{(\tau +1)}\,=\, & {} d\,\bigl \langle N \bigr \rangle - {\overline{n}}^{(\tau )}\;, \end{aligned}$$

5b $$\begin{aligned} \Delta {\overline{{\mathbf {x}}}}^{(\tau +1)}\,=\, & {} \bigl \langle \Delta {\mathbf {X}} \bigr \rangle + {\mathbb {C}}\text{ov}\bigl [{\mathbf {X}},N/\bigl \langle N \bigr \rangle \bigr ]\;, \end{aligned}$$

where the mapping for the fractions, written here in vector notation, holds for each strain *i* individually. It follows from the definition ${\mathbb {C}}\text{ov}\bigl [X_i,N\bigr ] = \bigl \langle NX_i \bigr \rangle -\bigl \langle N \bigr \rangle \bigl \langle X_i \bigr \rangle$ and the mean value ${\overline{x}}_i^{(\tau +1)} = \bigl \langle NX_i \bigr \rangle /\bigl \langle N \bigr \rangle$. Eq. (5b) is a version of the *Price equation* [49–51], usually describing how the frequency of a trait changes due to its inherent transmission bias and its covariance with fitness [41]. Here, the two terms have a clear interpretation in the context of multilevel selection: The average change of the population composition $\bigl \langle \Delta X_i \bigr \rangle$, indicates the difference between beginning and end of growth phase for strain *i*, averaged over inoculum sizes in all demes. It is driven by selection among cells growing in a single deme, and reflects local competition. Typically, it will be positive for strains with faster growth rates. The second term ${\mathbb {C}}\text{ov}\bigl [X_i,N/\bigl \langle N \bigr \rangle \bigr ]$ indicates selection among different demes in the mixing and reseeding phase, where large final population sizes are highly represented in the pool and therefore in re-seeding.

**Fig. 2:**
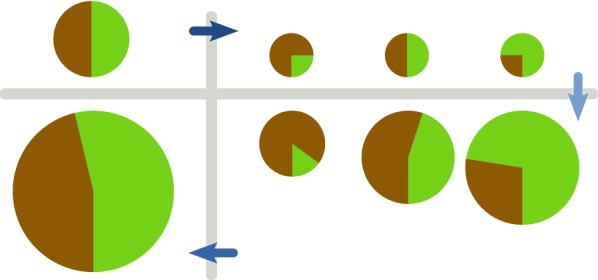
’Simpson’s paradox’ visualized in our model. Equal populations of two strains (top left) are seeded to demes with variable proportions (top right). At the end of the growth phase, the green strain has decreased in frequency in each deme (bottom right). Nevertheless because its frequency covaries with final population size, the green strain increases in frequency after pooling (bottom left)

Equation (5b) allows to study an effect known as *Simpson’s paradox* [52, 53]. It describes counter-intuitive statistical observations that arise due to structure of the underlying data. The illustration in Fig. 2 depicts this effect in the context of our model: The ’green’ strain looses in the local competition and declines in frequency over the growth phase, $\bigl \langle \Delta X_\text{green} \bigr \rangle < 0$. In the depicted example the inequality $\Delta X_\text{green}<0$ even holds for each group individually. However, a larger initial fraction of this strain correlates with a larger final size, ${\mathbb {C}}\text{ov}\bigl [X_\text{green},N/\bigl \langle N \bigr \rangle \bigr ] > 0$. If this correlation is strong enough, the sum of both effects will cause an increased frequency over cycles, $\Delta {\overline{x}}_\text{green}^{(\tau +1)} > 0$. This effect has been examined, for instance, in synthetic-biology experiments with microbial populations [54, 55].

## Results

### Analysis of isoclines in the cycle mapping

The general mapping of Eq. (5) will be analyzed in terms of its isoclines and fixed points, providing information on the conditions for takeover, coexistence, total extinction, and other possible outcomes of interacting populations. Isoclines are curves in phase space satisfying either $\Delta {\overline{n}}= 0$ or $\Delta {\overline{x}}_i =0$, such that one variable—the total inoculum size or the fraction of strain *i* – remains unchanged under the mapping. An isocline separates the space between regions where the variable increases or decreases. Fixed points of the dynamics appear at the intersections of all isoclines, where $\Delta {\overline{n}}=0$ and $\Delta {\overline{x}}_i=0$ holds for all *i*. Our analysis involves deriving approximations for isoclines which help understand the structure of phase space.

Below we illustrate all our results with two strains ($i=1,2$). Then, the phase space is a two-dimensional plane $({\overline{n}},{\overline{x}}_1)$; the other fraction is determined by ${\overline{x}}_2=1-{\overline{x}}_1$. Numerical algorithms for computing the isoclines are described in Additional file 1: Appendix S1.

### Indirect interaction by metabolic growth-yield trade-off

**Fig. 3:**
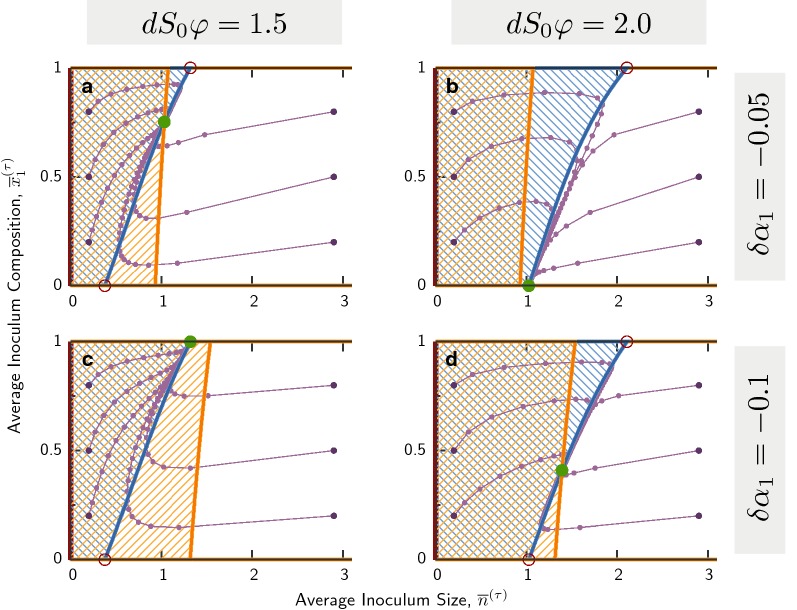
Phase plane for metabolic trade-off. Trajectories of average inoculum size ${\overline{n}}$ and average composition ${\overline{x}}_1$ are displayed in purple, where two connected dots indicate one cycle of growth, mixing and reseeding. Dark purple points indicate starting points for these trajectories, which are followed for 50 cycles, and can end in stable fixed points (full green circles). Empty red circles are unstable fixed points. Isoclines for total population size, $\Delta {\overline{n}}^\star =0$, are shown in blue, while isoclines for the composition $\Delta {\overline{x}}_1^\star =0$, are shown in orange. Hatched regions indicate areas where either variable over one cycle, while on the other side of the isocline they decrease. Parameters not stated in panels are $\delta \varphi _1 = 0.2$, $S_0\varphi =10^5$, $\alpha T_\text{mix}=24$

First, we carry out the isocline analysis for a very simple indirect interaction between two strains with constant growth rate and yield, and with a metabolic trade-off: strain 1 grows slower but is more efficient ($\delta \alpha _1 < 0$ and $\delta \varphi _1>0$). The phase plane $({\overline{n}},{\overline{x}}_1)$ is shown in Fig. 3 for four parameter sets. Sample trajectories of inoculum sizes in the cycle mapping are shown as connected purple dots starting from dark purple on the outside; all of them converge to stable fixed points (green circles) after few cycles. Unstable fixed points are depicted by red empty circles; trajectories pass near these points but are not attracted to them. Isoclines of total population size are drawn with *blue* lines, while isoclines of fraction with *orange* lines.

In panels A and D the stable fixed point is inside the plane, where $0\!<\!{\overline{x}}_1\!<\!1$, indicating coexistence between the two strains. In contrast, in panel B the fixed point is on the boundary ${\overline{x}}_1=0$ and in panel C on the boundary ${\overline{x}}_1=1$. These two cases represent fixation of one strain. Approximations for the isoclines are derived by solving the within-deme dynamics of Eq. (1), and then inserting the solutions into the cycle mapping of Eq. (5) (see Additional file 1: Appendix S1):

6a $$\begin{aligned} \Delta {\overline{n}}^\star =0\Leftrightarrow & {} {\overline{n}}^\star \approx dS_0\varphi \bigl (1 + \delta \varphi _1(2{\overline{x}}_1-1)\bigr )\; \end{aligned}$$

6b $$\begin{aligned} \Delta {\overline{x}}_1^\star =0\Leftrightarrow \, & {} {\overline{n}}\approx \frac{\vert \delta \varphi _1/\delta \alpha _1\vert }{\log (S_0\varphi )}\;. \end{aligned}$$

The position ${\overline{n}}^\star$ of the population size isocline in Eq. (6a) is influenced by a balance between the dilution rate *d* during mixing, and the increase in cell number of during growth, $S_0\varphi$, setting an equilibrium inoculum size given by the product $dS_0\varphi$. For a uniform population growing under cycles, ${\overline{n}}^\star = dS_0\varphi$ is the vertical isocline of population size; we call this the *dilution line*. Larger inoculum sizes cannot be sustained over long times, and smaller ones will rapidly grow to this value and remain unchanged on average over cycles. With only two populations, the trade-off, $\delta \alpha _1 < 0$ and $\delta \varphi _1 > 0$, rotates the dilution line by a positive angle from its vertical position (Fig. 3). Trajectories in all figure panels converge quickly to this tilted isocline, implying that total population size equilibrates faster than composition [56, 57]. We shall see that this tilted dilution line, as well as the separation of time-scales between total population size and its composition also occur also in other interactions analyzed below.

Increasing $d S_0 \varphi$ shifts the tilted dilution line to higher ${\overline{n}}$ while leaving its general shape almost invariant (going from panels A, C to B, D). In contrast, this parameter has negligible influence on the composition isocline, which depends strongly on $\delta \alpha _i$. The latter has the form ${\overline{n}}\approx const$, which is independent of ${\overline{x}}_1$, and thus appears as a(n almost) vertical line in phase space.

**Fig. 4:**
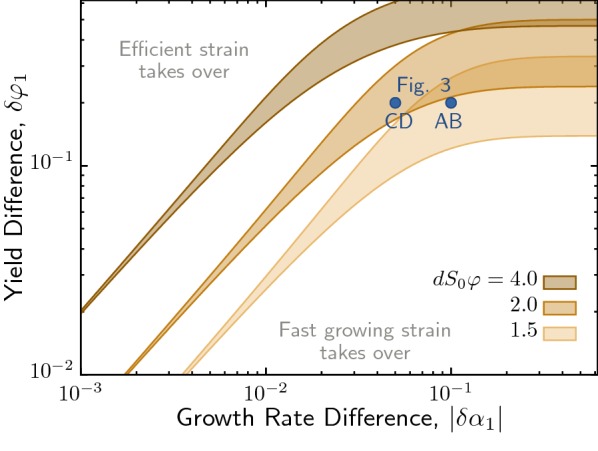
Coexistence regions for metabolic trade-off. Long-term outcome of mapping over cycles in parameter space representing the metabolic trade-off ($\delta \alpha _1,\delta \varphi _1$). Shaded regions indicate stable coexistence, outside this region one of the strains will take over. Parameter values indicated by blue dots correspond to the panels in Fig. 3. Depending on the dilution rate *d*, these parameters are either inside or outside the coexistence region

We have seen that isoclines can intersect inside the phase plane to generate a stable coexistence fixed point. Now we ask what the conditions on the metabolic trade-off are for such coexistence? In Fig. 4 we show the area in parameter space $(\delta \alpha _1,\delta \varphi _1)$ supporting coexistence. This reveals two distinct regions of different behavior: In the large trade-off regime ($\vert \delta \alpha _1\vert \sim {\mathcal {O}}(10^{-1})$), broad areas of coexistence appear as strips almost independent of $\delta \alpha _1$. There, the growth rate difference is large enough that all demes are dominated by the fast growing strain when seeded by a mixture. Coexistence arises only due to demes that were seeded with the efficient strain alone, and it can grow without local competition to large final sizes. The degree of efficiency trade-off, $\delta \varphi _1$, supporting this coexistence depends on the equilibrium population size parameter $dS_0\varphi$. For small trade-off regime, in contrast, the parameter range supporting coexistence is narrow and depends on a delicate balance between $\delta \alpha _1$ and $\delta \varphi _1$. Here, demes exhibit an almost continuous spectrum of possible final sizes at mixing, compared to the essentially only two outcomes in the previous regime. In this case, coexistence is much less robust and requires fine-tuning of the trade-off.

### Public good interactions

Public goods are extracellular products that promote or enhance growth, and are usually available to all cells within a shared environment. Often, public goods are actively produced by cells and generate ecological interactions through the environment. In well-mixed environments, public goods can lead to the ’tragedy of commons’ [58], where producing (sub-)populations that pay a cost for production of the public good are out-competed and go extinct. In spatially structured environments this outcome can be averted, in particular when segregation supports Simpson’s paradox (Fig. 2). In the following, we analyze two classes of public good interactions: One is the collective resistance to antibiotics, mediated by secretion of an antibiotics-hydrolyzing enzyme. The second involves active extraction of a growth resource from the environment, specifically iron-chelation by extracellular pyoverdine. These two examples lead to a time-dependent growth rate $\alpha _i(t)$ or a time-dependent yield $\varphi _i(t)$, allowing us to compare the effect of spatio-temporal structuring in two types of interactions with different costs and benefits. In order to couple these dynamics to our spatio-temporal model, we add one equation describing the public goods dynamics to the within-deme growth, Eq. (1), which then influences either $\alpha _i(t)$ or $\varphi _i(t)$.

#### Collective reduction of antibiotics

Extracellular enzymes that reduce the concentration of antibiotics in the environment can be considered a prime example for a public good. We model a scenario where an antibiotic with a concentration $B(0) = B_0$ is supplemented at the seeding step in all demes. The entire population may be able to recover and grow, if at least one strain can reduce this concentration over time, potentially leading to cross-protection [59, 60]. It has been reported that the effectiveness to treat bacterial populations with antibiotics depends the initial population density [61–64], which will be crucial to determine the outcome of the within-deme dynamics.

In the following, we assume only one of two strain produces and secretes enzymes that hydrolyze antibiotics. We assume that this comes at a cost in terms of growth rate [54, 65], such that the producing strain has a lower growth rate ($\delta \alpha _1<0$), while yield is assumed to be identical ($\delta \varphi _1=0$). Antibiotics-induced death is coupled to metabolic processes, such that death rate is proportional to growth rate in the absence of antibiotics [66, 67]. With these considerations, a common model for the effective growth rate of microbial populations exposed to antibiotics is [68],

7 $$\begin{aligned} \alpha _i(t) = \alpha \bigl (1+\delta \alpha _i\bigr )\frac{1-\bigl (B(t)/\mu \bigr )^\kappa }{1+\bigl (B(t)/\mu \bigr )^\kappa /\gamma }\;. \end{aligned}$$

Here, the fraction in the last term—indicating the time-dependent function *A*(*t*)—is a sigmoidal Hill-function. While without antibiotics we had $A(t)=1$, here *A*(*t*) decreases with increasing antibiotic concentration: At the ’minimal inhibitory concentration’ (MIC) of $B(t)/\mu =1$ population growth it becomes zero and switches sign to population death. For large antibiotic concentrations, the time-dependent term saturates at $A(t) = -\gamma$. The steepness of *A*(*t*) around the MIC is determined by $\kappa$. Compared to this dramatic effect of antibiotics on growth rate, we assume that growth rate differences $\delta \alpha _i$ are small.

The changing antibiotic concentration affects growth rates of all inhabiting populations simultaneously. The concentration is reduced by the production strain,

8 $$\begin{aligned} \dot{B} = -\rho _1N_1B\;, \end{aligned}$$

where $\rho _1$ characterizes the rate of resistance of strain 1, incorporating both expression rate of hydrolyzing enzymes and efficiency of their degradation reaction. This equation for *B*(*t*) is added to Eq. (1) which in turn affects both growth rates $\alpha _i(t)$.

Coupling these interactions—how antibiotics changes the growth rates in a deme, Eq. (7), and how the its concentration is reduced, Eq. (8)—generates a race between two processes. Either the amount of antibiotics is large enough to kill all cells within a single deme, or microbes can reduce the concentration below $B(t)/\mu < 1$ in time for the population to recover. For $T_\text{mix}$ long enough, such that recovering populations deplete all nutrients, we will find either a fully grown population or no cells at all.

**Fig. 5:**
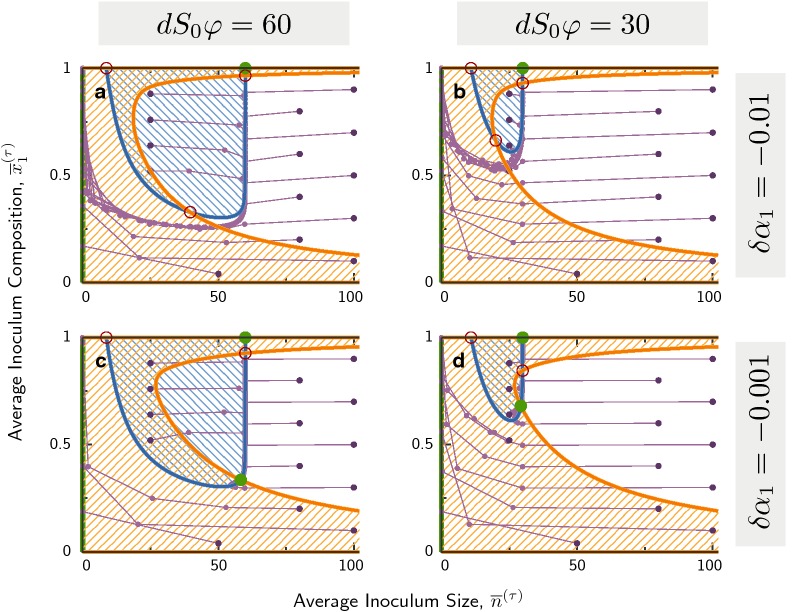
Phase plane for public goods interaction: collective antibiotic resistance. Trajectories are shown in purple, starting at dark purple points. Strain 1 produces an antibiotic-degrading enzyme at the cost of a slower growth rate. Blue and orange lines indicate the isoclines for total population size and composition. Blue and orange hatched areas indicate an increase in the corresponding variable. Parameters not indicated in the figure: $B_0/\mu =1.25$, $\rho _1/\alpha = 5\cdot 10^{-3}$, $S_0\varphi =10^5$, $\kappa =\gamma =2$

Figure  5 shows four examples of phase planes with the isoclines and trajectories marked as before. Approximating the within-deme dynamics, the shapes of the two isoclines are determined by

9a $$\begin{aligned} \Delta {\overline{n}}^\star =\, 0\Leftrightarrow \, & {} {\overline{n}}^\star \approx \left\{ \begin{array}{l}dS_0\varphi \\ \frac{1}{{\overline{x}}_1}\,\frac{\kappa \gamma \alpha _1}{(1+\gamma )\rho _1}(\log B_0/\mu )^2 \end{array}\right. \;, \end{aligned}$$

9b $$\begin{aligned} \Delta {\overline{x}}_1^\star = 0\Leftrightarrow\, & {} {\overline{n}}\sim \left\{ \begin{array}{l} \frac{1}{1-{\overline{x}}_1^\star }\\ \frac{1}{\delta \alpha _1}\frac{1}{({\overline{x}}_1^\star )^{1+\epsilon }} \end{array}\right. \;. \end{aligned}$$

The population size isocline for antibiotic reduction, Eq. (9a) (blue line in Fig. 5), features two distinct connected parts: One is the dilution line ${\overline{n}}^\star \approx dS_0\varphi$, implied by the resource limitation on population size already found before. This limitation determines equilibrium population size for large inoculum sizes and large fractions of the resistant strain ${\overline{x}}_1$ that defines a region where antibiotics are easily reduced. In contrast, survival for small inoculum sizes depends on having enough producing cells to reduce antibiotics in time to prevent extinction. This defines a threshold on ${\overline{n}}_1={\overline{n}}\,{\overline{x}}_1$, and hence the isocline scales as ${\overline{n}}^\star \sim 1/{\overline{x}}_1$, creating its antibiotics-limited part. The blue hatched area enclosed by these two parts shows increasing inoculum sizes between cycles, and thus can lead to survival in the long run.

The composition isocline (orange line in Fig. 5) also exhibits two parts: In the region of large ${\overline{x}}_1$, resistant cells succeed in reducing the antibiotics below $B(t)/\mu = 1$ in time to reverse death into growth. However, during this time both strains are dying. In order for the non-producers to survive this phase, they need to exceed a threshold in their inoculum size ${\overline{n}}_2$, which leads to the top scaling of the isocline, ${\overline{n}}_2={\overline{n}}(1-{\overline{x}}_1)=const$. Above this isocline the producing strain fixates and trajectories flow to the stable fixed point ${\overline{x}}_1=1$ (green circle on top boundary, present in all panels).

The scaling for smaller ${\overline{x}}_1$ results from a balance between two opposing effects: Populations that have a larger initial fraction $x_1$ are more likely to overcome the antibiotic threat and survive, thus the mean ${\overline{x}}_1$ increases for the next cycle. These gains are offset against the losses in local competition, which are of order $\delta \alpha _1$. Details are described in Appendix S1, where we derive the scaling of the isocline reported in Eq. (9b), where $\epsilon$ a small empirical positive constant.

It is interesting to note that, similar to the simple resource consumption case, the two isoclines are largely determined by two parameters independently: $dS_0\varphi$ governs the position of the population isocline, whereas growth rate difference $\delta \alpha _1$ governs the position of the composition isocline. Each of these parameters has a negligible effect on the other isocline.

We have seen that, for large enough fraction of producer cells, the population isocline intersects with the top boundary to form an all-producer stable fixed point. An important question is, under what conditions can the two strains coexist over long times? Such coexistence implies cross-protection due to the spatio-temporal structure, as non-producing cells manage to “hitch-hike” to the next cycle and survive. Figure 5 shows that if the resistant strain suffers a serious growth-rate cost, it will not be able to cross-protect the sensitive strain and carry it over multiple cycles (A, B). In this case, the isoclines cross at an unstable fixed point and the only stable outcomes are complete extinction or fixation of the resistant strain. However, if this cost is not too high (C, D), stable coexistence can arise.

**Fig. 6:**
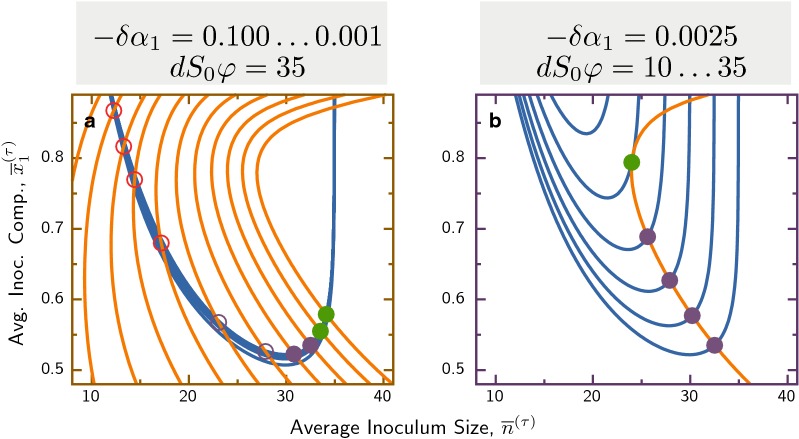
Zoom onto isocline intersections with varying parameters. **a** Varying growth rate difference, $\delta \alpha _1 = 10^{-1}\dots 10^{-3}$, shifts the composition isocline (orange lines), but leaves the population size isocline (blue lines) almost unaffected: The fixed point is unstable as long as the intersection is on the antibiotics-limited part of the population isocline (red open circles), and becomes stable when the intersection moves to the resource-limited part (green filled circles). Intersections close to the transition have complex eigenvalues, which can be stable (purple filled circles) or unstable (purple open circles). The unstable fixed point can support a stable limit cycle (purple open circles), not contained in the linearized analysis. **b** Increasing the dilution rate shifts the population size isocline. Parameters in both panels are $B_0/\mu =1.25$ and $\rho _1/\alpha =5\cdot 10^{-3}$, $\kappa =\gamma =2$

The shapes of the isoclines reveal a simple condition for the stability of coexistence, depending on the position of their intersection: If they intersect on the antibiotics-limited branch of the population size isocline (left part), the coexistence fixed point is unstable. If this intersection occurs on the resource limited dilution line (right part), it is stable. The direction of flow in the phase plane dictates that, in the former case, trajectories arriving from the right with increasing ${\overline{x}}_1$ are already in the no-survival regime (outside the hatched blue region) and thus must flow to extinction. In contrast, in the latter cases these trajectories can potentially arrive to the intersection. In Fig. 6 we explore how the stability of this fixed point changes, when varying either of the two parameters governing the isoclines. Interestingly, at the transition from stable to unstable fixed point a limit cycle can occur via a Neimark-Sacker bifurcation [69], which is the analogue of a Hopf bifurcation in the discrete dynamics of the cycle mapping. In this case, both average size and composition of demes oscillate over multiple cycles (see Appendix S1).

#### Iron extraction via siderophores

A different kind of indirect interaction occurs when a growth-promoting substrate is actively extracted from the environment by extracellular products. Such a scenario is generated by pyoverdine, an iron-chelating siderophore produced by several *Pseudomonas* species. Pyoverdine strongly binds otherwise almost unavailable iron, and allows microbes to uptake the iron-pyoverdine complex via special transport proteins. Siderophores have often been considered to be a public good [55, 70–72], but more recent experiments showed that this classification is highly dependent on details of environmental conditions [73, 74]. Physiologically, enhanced iron-availability seems to increase the yield of cells [75, 76], as populations grow to larger size with the same amount of nutrients. As before, we analyze the interaction between a producing and non-producing strain in the spatio-temporally structured environment. This provides an example of time-dependent yields induced by the varying level of obtainable iron from the environment.

While the exact relation between yield and siderophore concentration is hard to specify, a few principles can guide our modeling: First, since cells require only minuscule quantities of iron and almost all experimental system will likely contain small traces of it, we assume they can maintain a minimal level of growth even without pyoverdine. Moreover, the effect of pyoverdine on yield saturates, with a maximum increase by a factor $\sigma$. We assume pyoverdine is shared among all cells within a single deme, which requires matching transport proteins [74, 77]. Taking these considerations together, we propose

10 $$\begin{aligned} \varphi _i(t) = \varphi \bigl (1+\delta \varphi _i\bigr )\left( \sigma - (\sigma -1)e^{-P(t)}\right) \;, \end{aligned}$$

which indicates an exponential convergence towards a maximal value of yield with increasing pyoverdine concentrations *P*. As before, each strain *i* is characterized by its fixed deviation in yield $\delta \varphi _i$, while the public good concentration *P*(*t*) affects all strains in the same time-varying way. For the dynamics of pyoverdine, *P*(*t*), we assume again that only strain 1 produces it,

11 $$\begin{aligned} \dot{P} = \rho _1N_1\;. \end{aligned}$$

The rate $\rho _1$ includes expression rate, excretion rate and the magnitude of their effect on yield, such that *P* itself is a dimensionless quantity, that can be used in the exponential function of Eq. (10).

Analyzing the system involves once again integrating the population and resource dynamics, Eq. (1), together with the pyoverdine concentration dynamics, Eq. (11). These solutions are inserted into the cycle map, Eq. (5), from which we can obtain the scaling for the two isoclines,

12a $$\begin{aligned} \Delta {\overline{n}}^\star\Leftrightarrow\, & {} {\overline{n}}^\star \approx \left\{ \begin{array}{l}dS_0\varphi \sigma \\ dS_0\varphi \end{array}\right. \;, \end{aligned}$$

12b $$\begin{aligned} \Delta {\overline{x}}_1^\star\Leftrightarrow \,& {} {\overline{n}}\sim \frac{\log \vert \delta \alpha _1\vert +C}{{\overline{x}}_1^\star }\;. \end{aligned}$$

**Fig. 7:**
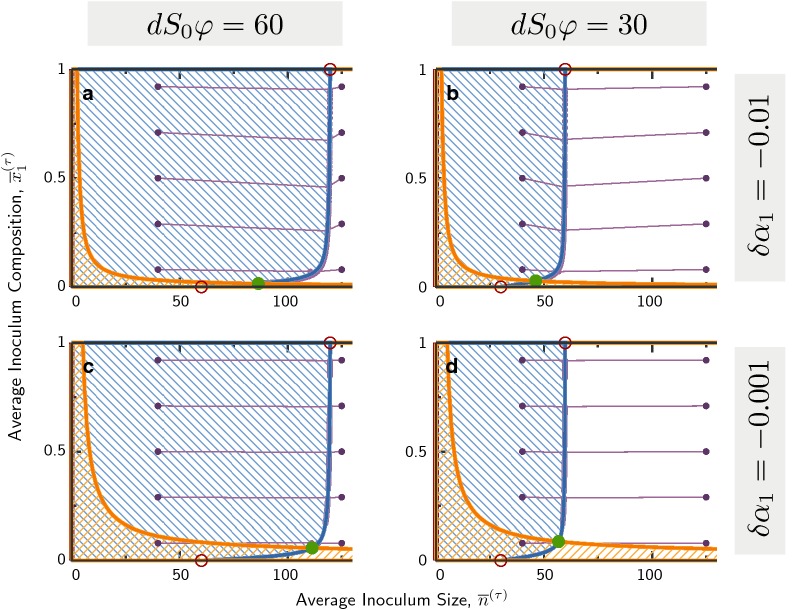
Dynamics over multiple cycles with production of pyoverdine that enhances iron-availability. Blue hatched areas indicate an increase of the average inoculum size ${\overline{n}}$ over one cycle, while orange hatched areas indicate an increase in the population composition ${\overline{x}}_1$. Strain 1 (${\overline{x}}_1=1$) differs from Strain 2 (${\overline{x}}_1=0$) by a slower growth rate $\delta \alpha _1<0$ and a non-zero production of pyoverdine $\rho _1>0$, $\rho _2=0$. Purple dots connected by lines show exemplaric trajectories, that lead to coexistence fixed points for the chosen parameters. Only the first 50 cycles from each trajectory are shown. Adjustment of the average inoculum size ${\overline{n}}$ is fast, while the population composition ${\overline{x}}_1$ changes slow. Growth rate differences are chosen to be $\delta \alpha _1 = -10^{-2}$ (**a**, **b**) and $\delta \alpha _1=-10^{-3}$ (**c**, **d**). Dilution rates (indicated by average inoculum sizes for growing populations) are $dS_0\varphi = 60$ (**a**, **c**) and $dS_0\varphi =30$ (**b**, **d**). Other parameters are $\sigma = 2$ and $\rho _1/\alpha = 10^{-3}$ for all panels

Without pyoverdine we would recover the dilution line; indeed this is where the isocline intersects with the boundary ${\overline{x}}_1=0$. Moving away from this boundary the isocline becomes a dilution line enhanced by a factor $\sigma$, indicating saturated pyoverdine concentration, see Fig. 7.

The approximation for the composition isocline is obtained using numerical evaluation of the terms, and is described in Additional file 1: Appendix S1. The $({\overline{x}}_1^\star )^{-1}$ scaling is important for coexistence: This hyperbolic shape typically intersects the (vertical) isocline for population size. Thus, the stable long-term outcome in the cycle dynamics is maintenance of a small producer population, whose coexistence with a non-producing strain is indicative of cooperation.

## Discussion

In this article, we investigated various types of interactions between growing microbial populations that are repeatedly mixed and separated into compartmentalized demes on a long time-scale. These interactions include growth on a shared resource with a metabolic trade-off, which was extended by two specific examples of cooperative interactions: antibiotic degradation to allow growth, or enhancing iron availability by a chelator. In both cases extra-cellular products, also known as public goods, are secreted and affect all cells. The reduced growth rate of the producing strains would therefore result in fixation of faster non-producing strains in a homogeneous environment. We investigate how this outcome changes in a spatio-temporally structured environment in our model and how it depends on details of the interaction. We found that the spatio-temporal structure, together with the variance of initial conditions for the growth processes, commonly allows costly cooperative traits to stably coexist over multiple cycles of seeding, growth and mixing.

By formulating the dynamics in terms of fractions and total population size, we found that the population composition ${\overline{{\mathbf {x}}}}$ obeys the Price equation [41, 49], $\Delta {\overline{{\mathbf {x}}}}^{(\tau +1)} = \bigl \langle \Delta {\mathbf {X}} \bigr \rangle + {\mathbb {C}}\text{ov}\bigl [\mathbf{X},N/\bigl \langle N \bigr \rangle \bigr ]$ (see Eq. (5b)). This is in agreement with previous work on trait-group-models [35, 37–39]. The ecological life cycle imposed externally on the population in our model, lets us interpret these two terms directly as the two levels of selection: $\bigl \langle \Delta {\mathbf {X}} \bigr \rangle$ is the average change in the within-deme dynamics, usually dominated by fast growth. The second term, ${\mathbb {C}}\text{ov}\bigl [\mathbf{X},N/\bigl \langle N \bigr \rangle \bigr ]$, indicates correlations of the population composition with (relative) total population size, and thus describes selection between populations of different demes. In this context, ’Simpson’s paradox’ occurs when the first term is negative, but the second covariance term is positive and large enough to make the the total change positive.

However, the Price equation only models frequency dynamics, while previous work has highlighted the importance of population sizes as well [11, 78]. In our framework, population size is a dynamic variable, kept finite by including also growth resource as a dynamic variable. Importantly, this finite population size at the end of growth is variable and depends on conditions in each deme. Thus population size takes the center stage, as it plays the role usually occupied by fitness. If an inoculum generates a larger final population size—due to reduction of antibiotics, enhanced iron-availability, or just more efficient resource conversion – then populations from such demes will be over-represented in the next cycle. Diluting the pool by a constant *factor* after resource depletion, rather than re-seeding by a fixed number, allows the inoculum size to vary according to the outcome of the previous growth phase. This effectively carries over the representation in the pool to the next generation, upon which selection can act.

We note that another possibility for modeling finite populations is to introduce logistic growth. This approach describes in an abstract manner many factors potentially limiting growth, including finite resources. We have chosen the more direct approach of explicit resource dynamics [79–81], which clarifies the distinction between strains with different efficiencies in utilizing this resource for growth. We note that this differs from early ecological models with an implicit limitation via carrying capacity, known as ’r/K-selection’ [82].

With population size and composition as explicit dynamic variables, we employed phase-plane analysis to study several instances of interactions between strains that vary in their intrinsic properties. These instances all implement some cooperative interaction between strains, but differ in their kinetics and biological detail. An important tool for this is the analysis of population size and frequency isoclines, their geometry, parameter dependence, and how they constrain the system trajectories. This analysis characterizes the long-term outcomes of the system, which include extinction, exclusion (single-strain) fixed-points and coexistence fixed-points. We found that all instances of cooperative interactions could support long-term coexistence in the spatio-temporally structured habitat.

Regardless of the type of interaction, total inoculum sizes equilibrate rapidly to a value determined by environmental conditions, while changes in composition occur much more slowly. Such a separation of time-scales is known for other models as well [56, 57]. An interesting observation is that each variable is governed by a different parameter of the system. The dilution rate, which sets the position of the inoculum size isocline, can be chosen independent from growth rate differences, which influences the composition isocline. Thus, as both isoclines can be shifted independently in phase space, intersections that indicate coexistence are obtainable for many parameter values.

Cooperative interactions between microbial strains have often been described by game theoretical approaches [83–85]. This simplifies the potential complexity of the system and can classify different types of interactions. Here we have used a dynamical-systems which incorporates more biophysical ingredients. This opens the possibility for a richer spectrum of outcomes and takes into account also dynamic effects. For example in the case of antibiotic resistance, both population survival and coexistence depend on rates of competing processes and on initial conditions.

An application of concepts similar to the trait-group-models [35, 38] to microbial populations was previously explored for instance in [10–13]. There, the authors deal with finite population sizes in segregated demes, fluctuations during the initial time of growth, and a second, long time-scale on which all populations are mixed repeatedly. These fluctuations in cell numbers during initial stages of growth amplify benefits to the whole population, lead to larger final sizes, and are thus important for coexistence. That fluctuations can leave traces in the population composition long after this initial time is known for a long time in principle [86], and has also been applied to microbial populations [87, 88]. Our model contains this effect as well, although in a simplified form: variation only occurs in the inoculum, and this variation is a crucial feature even though we mostly focus on mean values. Ultimately, such initial fluctuations provide the variation on which natural selection can act on the long time-scale.

In our model, we did not consider cell death to be an important contribution to the within-deme dynamics. This is a reasonable approximation for microbial populations, where growth often happens within hours, but significant decay only sets in on the time-scale of multiple days. However, this limits our model such that $T_\text{mix}$ needs to be roughly the same magnitude as $T_\text{depl}$ to be a realistic approximation. For approaches that include death in the dynamics, choosing the mixing time becomes more important [12, 89, 90], and is then a crucial parameter that determines if social traits are selected. If mixing time is *very* long, completely different aspects of microbial population dynamics become relevant [91, 92].

We have modeled a *perfect separation* of demes during growth, which are then instantly and globally mixed after a *constant* mixing time $T_\text{mix}$. What would happen, if any of our modeling assumptions were subject to uncertainties? One way to weaken the assumption of separated demes is to investigate populations in continuous space with limited dispersal. In such a setting, it was found that coexistence emerges on intermediary diffusion rates [93, 94]: Very fast diffusion makes the spatial dependence disappear altogether, while too slow diffusion leads to extinction of non-producers. A similar effect can be found in models of social evolution, which treat ’viscous populations’ [95, 96] with limited dispersal, but not the fully discrete structure of multiple populations in demes. Recently, several groups have tried to model the effects of specific interactions as a directed graph [97–100], where—depending on topology of interactions—fixation of alleles could either be enhanced or hindered. While most of these analyses treat each of the interacting nodes as individuals, we expect that if each of them encompasses full population dynamics on its own timescale, the spectrum of possible outcomes will also be skewed by the topology, that determines how populations migrate or are mixed. Finding answers to these issues would be one possible avenue for future work.

## Conclusion

In conclusion, we formulated and analyzed models of social interactions of spatially distributed microbial populations. Our results showed that coexistence—also of costly social traits—can be supported by the simple ecological mechanisms of a second time-scale and spatially distributed populations, two conditions that are arguably ubiquitous in nature. The dynamics of these traits can be described by an expression akin to the Price equation, which allowed reasoning within a framework that generalizes several previously published modeling approaches. Moreover, we also expect other collective dynamics to show similar behavior [101], when they are subject to similar spatio-temporal structuring of the environment.

## Supplementary information

**Additional file 1: Appendix S1.** Additional details of derivations for the different interaction mechanisms.

## Data Availability

Numerical code is available at https://github.com/lukasgeyrhofer/mixingcycles.
