# Supplementary material for: Coexistence and cooperation in structured habitats
Source: BMC Ecol. 2020 Mar 2;20:14. doi: 10.1186/s12898-020-00281-y (PMC7053132; doi:10.1186/s12898-020-00281-y)
Supplement: Supplementary file 1 — Additional file 1: Appendix S1. Additional details of derivations for the different interaction mechanisms. [file 12898_2020_281_MOESM1_ESM.pdf]

Coexistence and Cooperation in Structured Habitats

Appendix S1: Additional details of derivations

Lukas Geyrhofer* and Naama Brenner†

Network Biology Research Laboratories, and Department of Chemical Engineering, Technion – Israel Institute of Technology, Haifa, Israel

CONTENTS

S1.1. Growth dynamics of resource consumption 1
- A. General framework 1
- B. Dynamics of a single strain 2
- C. Dynamics of multiple strains 3
- D. Computing isoclines 4
- E. Coexistence of multiple strains 5

S1.2. Growth dynamics with antibiotics 6
- A. From dynamical interactions within demes to expansion factors 6
- B. Isoclines of the cycle mapping 7
- C. Stability of the coexistence fixed point 8
- D. Additional parameter dependence of the cycle mapping 9

S1.3. Growth dynamics with pyoverdine 9
- A. From dynamical interactions within demes to expansion factors 9
- B. Computing the shape of isoclines 10
- C. Other parameter dependence 11

S1.4. Numerical methods 11

References 11

S1.1. GROWTH DYNAMICS OF RESOURCE CONSUMPTION

In this appendix, we present additional technical details for the resource consumption dynamics to justify the expressions stated in the main text. This is first done by deriving a general framework for coupling the within-deme dynamics to the cycle mapping. Specifically, this is achieved with the introduction of an "expansion factor" ξ, for which we derive approximations for each of the within-deme dynamics. The extensions to the interactions including either antibiotic reduction or enhanced iron-availability from pyoverdine are treated in their own appendices S1.2 and S1.3 below.

* l.geyrhofer@campus.technion.ac.il

† nbrenner@technion.ac.il

A. General framework

First, we restate the dynamics already disclosed in the main text for reference: Within a single deme we model the growth dynamics of multiple strains $N_i(t)$ and the decrease of the resource concentration $S(t)$:

$$\partial_t N_i = \alpha_i N_i \,, \qquad \text{(S1.1a)}$$

$$\partial_t S = -\sum_i \frac{\alpha_i N_i}{\varphi_i} \,, \qquad \text{(S1.1b)}$$

where ∂_t is a time derivative, and different strains i are characterized by their growth rates α_i and yields φ_i. These growth rates $\alpha_i(t)$ and yields $\varphi_i(t)$ will be separated into three terms,

$$\alpha_i(t) = \alpha\big(1+\delta\alpha_i\big)A(t) \,, \qquad \text{(S1.2a)}$$

$$\varphi_i(t) = \varphi\big(1+\delta\varphi_i\big)Y(t) \,. \qquad \text{(S1.2b)}$$

Here, α is the average growth rate and φ the average yield, computed as arithmetic mean over all strains. The deviations from these average values for individual strains are denoted by $\delta\alpha_i$ and $\delta\varphi_i$. With this definition, the average growth rate α can be used as unit of time αt, and the average yield acts as unit for substrate concentrations $S\varphi$, which then counts potentially growing cells. In addition, we collect all time-dependencies in the terms $A(t)$ and $Y(t)$. In the public good scenarios treated later, these time-dependent terms are used to couple to the public good dynamics. For this section that only treats resource consumption, we assume they are constant during the growth period, $A(t) = Y(t) = 1$. However, we set $A(t) = 0$ when resources are depleted within a deme, $S(t) = 0$ for $t > T_{\mathrm{depl}}$. Thus, the decreasing resource concentration due to growing populations in Eq. (S1.1b) acts as a timer to stop the dynamics.

We transform all population sizes $N_i(t)$ to total population size $N(t) = \sum_i N(t)$ and fractions $X_i(t) = N_i(t)/N(t)$ of different strains, which turns Eqs. (S1.1) into

$$\partial_t N = \alpha A(t) N\Big(1+\sum_i \delta\alpha_i X_i\Big) \,, \qquad \text{(S1.3a)}$$

$$\partial_t X_i = \alpha A(t)\,\delta\alpha_i X_i\Big(1-\sum_j \frac{\delta\alpha_j}{\delta\alpha_i}X_j\Big) \,, \qquad \text{(S1.3b)}$$

$$\partial_t S = -\frac{\alpha N}{\varphi}\frac{A(t)}{Y(t)}\sum_i \frac{1+\delta\alpha_i}{1+\delta\varphi_i}X_i \,. \qquad \text{(S1.3c)}$$

Eq. (S1.3b) describes the frequency changes in a multi-species Lotka-Volterra model, also known as replicator

dynamics, which has been studied extensively [2–4]. For us, however, the dynamics of the population size N is important as well, because interactions between microbial strains do not only change the frequency dynamics, but also how frequency dynamics couples to the population size dynamics.

Throughout our work, we assume that different strains are seeded into new demes by independent Poisson distributions, each with parameter $\overline{n}_i$ which is its mean size per deme in the pool:

$$\mathbb{P}\big[\mathbf{n}\big|\,\overline{\mathbf{n}}\big] = \prod_i \frac{\overline{n}_i^{n_i}}{n_i!}\exp\big(-\overline{n}_i\big) \; . \tag{S1.4}$$

Averages over all droplets using this Poisson distribution are written as angular brackets, such that average population sizes $\mathbf{N}$ are

$$\big\langle \mathbf{N} \big\rangle = \sum_{\mathbf{n}} \mathbb{P}\big[\mathbf{n}\big|\,\overline{\mathbf{n}}\big]\mathbf{N}(T_{\text{mix}};\mathbf{n}) \; . \tag{S1.5}$$

These averages are always evaluated at the mixing time T_{mix}, such that the global mixing step is represented exactly in this averaging. In some cases we also want to indicate the parameters of the seeding distribution directly: Then, we write the variables that we average over as arguments and the parameters after a vertical line, $\big\langle \mathbf{N}(\mathbf{n})\big|\,\overline{\mathbf{n}}^{(\tau)}\big\rangle$, which indicates that the average should be computed for the indicated variables with these parameters of the distribution.

As already stated in the main text, the changes in inoculum size and composition between cycles are given by

$$\Delta\overline{n}^{(\tau+1)} = d\big\langle N\big\rangle - \overline{n}^{(\tau)} \; , \tag{S1.6a}$$

$$\Delta\overline{\mathbf{x}}^{(\tau+1)} = \big\langle \Delta\mathbf{X}\big\rangle + \mathbb{C}\text{ov}\big[\mathbf{X}, N/\big\langle N\big\rangle\big] \; , \tag{S1.6b}$$

which is used here for reference within this appendix. In the following, we present solutions to the within-deme dynamics given by Eqs. (S1.3), insert them into this cycle mapping in Eqs. (S1.6), and then evaluate these expressions to obtain the isoclines, $\Delta\overline{n}^\star = 0$ and $\Delta\overline{x}_i^\star = 0$.

B. Dynamics of a single strain

At first we solve the within-deme dynamics for a single strain, with constant growth rate α and constant yield φ, feeding on a single finite resource, and without any other environmental interactions. This is the most basic model that fits our framework, and has the advantage that we can obtain analytic solutions that allow insight about the behavior of the system. With only a single train we can drop any index i for this section.

As long as cells are growing we find $N(t;n) = n\exp\big(\alpha t\big)$ as solution to Eq. (S1.1a). This expression can be used in the resource dynamics Eq. (S1.1b), which we integrate from $S(0) = S_0$ to $S(T_{\text{depl}}) = 0$: we obtain

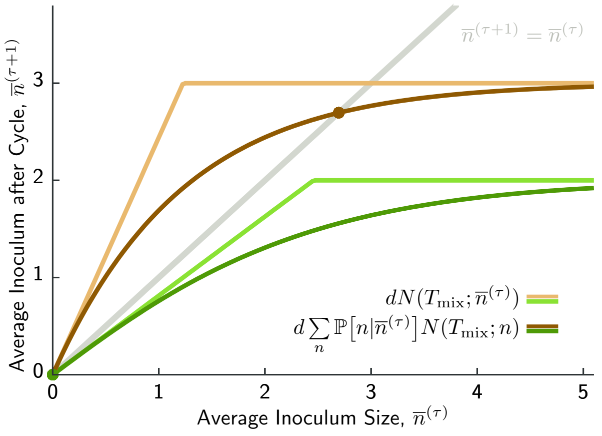

Figure S1.1. **Dynamics of a single strain with only resource consumption.** (A) Trajectories over time for two different inoculum sizes n and growth rates α_1. The population grows exponentially and then stays at constant numbers. (B) The inoculum size for a single is averaged with a Poisson distribution over all possible inoculum sizes. This smears the sharp transition from starting with enough cells that deplete all nutrients to inoculum sizes that are still in the exponential growth phase at T_{mix} (light colored thin lines). As explained in the text, existence of a fixed point depends on $dN(T_{\text{mix}};1) > 1$, and thus how many cells in the inoculum. If this condition is not met, the population will be washed out over multiple cycles.

$S_0 = \frac{n}{\varphi}\big(\exp(\alpha T_{\text{depl}}) - 1\big)$, which can be transformed to find the explicit depletion time T_{depl} as a function of n and the other parameters. Assuming that growth ceases upon resource depletion, $\alpha(t > T_{\text{depl}}) = 0$, the full solution for population size is

$$N(t;n) = \begin{cases} n\exp\big(\alpha t\big) \; , & 0 < t < T_{\text{depl}}(n) \; , \\ S_0\varphi + n \; , & T_{\text{depl}}(n) < t \; . \end{cases} \tag{S1.7}$$

In the dynamics over multiple cycles, this population sizes is evaluated at time T_{mix}. Thus, population size becomes a function of inoculum size n only. Depending on this number resources are either used up and final population size is given by the second expression, or the population is still in its growth phase and we need to use the first expression. These time-dependent solutions are shown in Fig. S1.1A, while panel B shows the mapping for inoculum sizes over cycles, and includes these solutions as function of n as light colored lines.

Explicitly, the mapping for the average inoculum size $\overline{n}^{(\tau)}$ takes the form

$$\begin{aligned}\overline{n}^{(\tau+1)} &= d\sum_{n=0}^{\infty}\mathbb{P}\big[n\big|\,\overline{n}^{(\tau)}\big]N(T_{\text{mix}};n) \\ &= d\sum_{n=0}^{n_{\text{thres}}(T_{\text{mix}})}\frac{(\overline{n}^{(\tau)})^n}{n!}e^{-\overline{n}^{(\tau)}}n\exp(\alpha T_{\text{mix}}) \\ &\quad + d\sum_{n=n_{\text{thres}}(T_{\text{mix}})+1}^{\infty}\frac{(\overline{n}^{(\tau)})^n}{n!}e^{-\overline{n}^{(\tau)}}\big(S_0\varphi + n\big)\end{aligned} \tag{S1.8}$$

In these expressions, the Poisson distribution smoothly interpolates the two branches of the population size in Eq. (S1.7), which is shown in Fig. S1.1B as darker solid lines.

This cycle mapping for a single strain has either one or two fixed points, but only one of them is stable: One fixed point always exists for empty inocula, $\overline{n}^\star = 0$, as cells cannot spontaneously grow out of nothing. If this is the only fixed point, extinction is a stable state of the dynamics. However, if a second fixed point exists, it will occur on the branch of the solution that indicates depleted resources, see Eq. (S1.7). This second fixed point with $\overline{n}^\star > 0$ is always stable when it exists, and extinction, $\overline{n}^\star = 0$, becomes an unstable fixed point upon appearance of this non-zero fixed point.

Stability of a fixed point in this one-dimensional mapping is determined by the slope of final population sizes with respect to the average inoculum size, $\partial_{\overline{n}}\langle N \,|\, \overline{n}\rangle|_{\overline{n}=\overline{n}^\star}$, evaluated at the fixed point $\overline{n}^\star$. If the absolute value of this expression is smaller than 1, the fixed point is stable, otherwise it is unstable. Before doing the calculation explicitly, however, we introduce an auxiliary step: taking a derivative with respect to the parameter of a Poisson distribution, we find

$$\partial_{\overline{n}}\mathbb{P}\big[n\big|\,\overline{n}\big] = \frac{\overline{n}^{n-1}}{(n-1)!}e^{-\overline{n}} - \frac{\overline{n}^{n}}{n!}e^{-\overline{n}} \,,$$

which simply leads to a shift in this summation index,

$$\partial_{\overline{n}}\big\langle F(n)\big|\,\overline{n}\big\rangle = \sum_{n=0}^{\infty}\mathbb{P}\big[n\big|\,\overline{n}\big]\big(F(n+1)-F(n)\big) \,, \quad \text{(S1.9)}$$

because we are only concerned with functions that exhibit the property $F(0) = 0$. This property just indicates that nothing grows in an empty inoculum. The expression (S1.9) generalizes straightforward to multidimensional variables, $\partial_{\overline{n}_i}\mathbb{P}\big[\mathbf{n}\big|\,\overline{\mathbf{n}}\big]$, where the shift occurs only for strain i.

With this calculation in place, the slope of the cycle mapping for a single strain at the origin, $\overline{n}^\star = 0$, is

$$\partial_{\overline{n}} d\big\langle N\big|\,\overline{n}\big\rangle|_{\overline{n}^\star=0} = dN(T_{\text{mix}};1) \,, \qquad \text{(S1.10)}$$

where we used the Poisson distribution for parameter $\overline{n} = 0$ $\mathbb{P}\big[n\big|\,0\big] = \delta_{n0}$. When nutrients are not yet depleted at mixing, the Eq. (S1.10) evaluates to $d\exp\big(\alpha T_{\text{mix}}\big)$. Thus, large dilution rates and/or long mixing times enable a stable fixed point, which exists when $d\exp\big(\alpha T_{\text{mix}}\big) > 1$. On the other hand, if nutrients are already depleted at mixing, the expression in Eq. (S1.10) simply evaluates to $dS_0\varphi$. Thus, depending on parameters, this extinction fixed point can be either stable or unstable.

Further analytic progress can be made with the assumption that mixing time is large enough that even a single cell uses up all nutrients. Then, the cycle mapping in Eq. (S1.8) only contains terms in the second sum, as $n_{\text{thres}}(T_{\text{mix}}) = 0$. This sum can be computed, and we find the mapping

$$\begin{aligned}\overline{n}^{(\tau+1)} &= d\big\langle N\big|\,\overline{n}^{(\tau)}\big\rangle \\ &= d\left(\big(1-e^{-\overline{n}^{(\tau)}}\big)S_0\varphi + \overline{n}^{(\tau)}\right) \,.\end{aligned}$$

From this expression, we can compute the fixed point $\overline{n}^\star$,

$$\begin{aligned}\overline{n}^\star &= \frac{dS_0\varphi}{1-d} + \mathscr{W}\left(-\frac{dS_0\varphi}{1-d}\exp\Big(-\frac{dS_0\varphi}{1-d}\Big)\right) \\ &\approx dS_0\varphi \,, \qquad \text{(S1.11)}\end{aligned}$$

where $\mathscr{W}$ is the Lambert-W function. For larger $dS_0\varphi$, the argument in $\mathscr{W}$ becomes very small, and $\mathscr{W}$ becomes linear in its argument, $\mathscr{W}(-y\exp(y)) \approx -y\exp(-y)$. This leads to an exponential correction for the single strain fixed point, $\overline{n}^\star \approx dS_0\varphi\big(1-\exp(-dS_0\varphi)\big)$, which can explain, for instance, why the population size isocline in Fig. 3 is shifted slightly off the value of the linear approximation in Eq. (S1.11).

Stability of this non-zero fixed point can be checked by

$$\begin{aligned}\partial_{\overline{n}} d\big\langle N\big|\,\overline{n}\big\rangle|_{\overline{n}^\star} &= d + dS_0\varphi e^{-\overline{n}^\star} \\ &\approx d + \overline{n}^\star e^{-\overline{n}^\star} \,, \qquad \text{(S1.12)}\end{aligned}$$

which is smaller than 1 for any realistic value of d, since the term involving the exponential is bounded from above by $1/e$. Thus, the fixed point given by Eq. (S1.11) will be stable. Its existence can be determined by the instability of the extinction fixed point at the origin, $\overline{n}^\star = 0$, which occurs for $dS_0\varphi \gtrapprox 1$.

C. Dynamics of multiple strains

The main technical challenge for multiple strains is to determine the depletion time $T_{\text{depl}}(\mathbf{n})$ for arbitrary inoculum vector $\mathbf{n}$. This was possible for a single strain, as shown in the previous section. In this section we will introduce the expansion factor ξ, that lets us circumvent this problem and allows progress with approximations.

First, we define this *average expansion factor* of a population as

$$\xi(t) \equiv \exp\Big(\alpha\int_0^t dt' A(t')\Big) \,. \qquad \text{(S1.13)}$$

In the special case of constant growth rate, $\alpha(t) = \alpha$, this reduces to $\xi(t) = \exp(\alpha t)$. Using ξ, the solutions to total size N and population fractions X_i in Eqs. (S1.3) are

$$N(t;n,\mathbf{x}) = n\xi(t)\Big(\sum_j x_j\xi(t)^{\delta\alpha_j}\Big) \,, \qquad \text{(S1.14a)}$$

$$X_i(t;n,\mathbf{x}) = x_i\xi(t)^{\delta\alpha_i}\Big/\Big(\sum_j x_j\xi(t)^{\delta\alpha_j}\Big) \quad \text{(S1.14b)}$$

with n and $\mathbf{x}$ the initial conditions for total population and fractions in a given deme. A time-dependence in these solutions enters only via the integration limit in $\xi(t)$. In Eqs. (S1.14) we also see the justification for the name "expansion factor": populations expand from initially n cells to the final numbers of roughly $n\xi$ cells, with corrections due to different growth rates.

One step towards obtaining solutions is to compute the expansion factor at the time of depletion, $\xi(T_{\text{depl}})$. Its derivation involves integrating resource use, Eq. (S1.3c), from $S(0) = S_0$ to $S(T_{\text{depl}}) = 0$, which gives

$$S_0\varphi Y(0) + \varphi \int_0^{T_{\text{depl}}} dt\ S(t)\big(\partial_t Y\big)(t)$$
$$= n\sum_i x_i \frac{\xi(T_{\text{depl}})^{1+\delta\alpha_i} - 1}{1+\delta\varphi_i}\ . \qquad \text{(S1.15)}$$

For time-constant yields, $Y(t) = 1$, and a sufficiently large amount of resources, such that $\xi \gg 1$, this relation simplifies: The integral with its explicit time-dependence drops, and T_{depl} only enters via the final value of the expansion factor $\xi(T_{\text{depl}})$. Consequently, Eq. (S1.15) reduces to

$$\frac{S_0\varphi}{n} \approx \xi \sum_i \frac{x_i \xi^{\delta\alpha_i}}{1+\delta\varphi_i}\ ,$$

which we can approximately solve as

$$\xi\big(T_{\text{depl}}(n,\mathbf{x})\big) \approx \frac{S_0\varphi}{n} G(n,\mathbf{x})\ , \qquad \text{(S1.16)}$$
$$G(n,\mathbf{x}) = 1 + \sum_i x_i\delta\varphi_i - \log\big(S_0\varphi/n\big)\sum_i x_i\delta\alpha_i\ .$$

For small differences between the strains, $\delta\alpha_i, \delta\varphi_i \ll 1$, we have $G \approx 1$ and thus the leading order is simply $\xi \approx S_0\varphi/n$.

The expansion factor $\xi(T_{\text{depl}})$ can be interpreted as a (non-linear, but monotonic) transformation of the depletion time T_{depl}. With this viewpoint demes with an increased yield relative to the average, $\sum_i x_i\delta\varphi_i > 0$, strains will grow longer (larger ξ), while in demes with growth faster than average, $\sum_i x_i\delta\alpha_i > 0$, the time to depletion decreases (smaller ξ). The weighted growth rate differences also feature a coefficient $\log(S_0\varphi/n)$, which reflects the fact that growth rate differences usually have a larger impact on the dynamics and can be easier (and faster) selected, compared to population level traits like yield and its differences between strains.

Since we assume that growth stops when resources are depleted, also the value of ξ stays constant after the depletion time T_{depl}. For the single strain case, we showed that a strain that does not deplete all nutrients when seeded alone, will ultimately go extinct. Thus, the relevant case is faster depletion than mixing, $T_{\text{depl}} < T_{\text{mix}}$, such that $\xi(T_{\text{mix}}) = \xi(T_{\text{depl}})$, and we will use the Eq. (S1.16) as $\xi(T_{\text{mix}})$.

The expansion factor will also be used later to mediate the coupling to additional public good dynamics. Influences of varying growth growth rates are already contained in the definition (S1.13) itself. Any changes in yield can be treated in the full resource-use equation (S1.15). The two scenarios with collective antibiotic resistance and iron-extraction via pyoverdine will be examples for either of these extensions.

D. Computing isoclines

To develop approximate expressions for the isoclines, we use the solutions of the within-deme dynamics, Eq. (S1.14), and insert them into the changes over cycles, Eq. (S1.6). Using a weak selection approximation ($\delta\alpha_i \ll 1$), we expand to first order in growth rate differences, $\mathcal{O}(\delta\alpha)$. Specifically, this will involve the approximation $\xi^{\delta\alpha_i} \approx 1 + \delta\alpha_i \log\xi$. For the change in total inoculum size, we obtain

$$\Delta\overline{n}^{(\tau+1)} = d\Big\langle n\xi\sum_i x_i\xi^{\delta\alpha_i}\Big\rangle - \overline{n}^{(\tau)} \qquad \text{(S1.17)}$$
$$\approx d\big\langle n\xi\big\rangle + d\sum_i \delta\alpha_i\big\langle x_i n\xi\log\xi\big\rangle - \overline{n}^{(\tau)}\ .$$

This is a general expression in terms of ξ that will also hold for public goods dynamics below. Inserting the approximation for ξ obtained specifically for resource consumption, Eq. (S1.16), is exactly the next step. the change in inoculum size is given by

$$\Delta\overline{n}^{(\tau+1)} = dS_0\varphi\Big(1 + \sum_i \overline{x}_i^{(\tau)}\delta\varphi_i\Big) - \overline{n}^{(\tau)}\ . \qquad \text{(S1.18)}$$

Thus, $\Delta\overline{n}^{(\tau+1)}$ becomes independent of growth rate differences (up to first order), as all terms in $\delta\alpha_i$ cancel with the dependencies contained in ξ, see Eq. (S1.13). To obtain the isocline equation, we set $\Delta\overline{n}^\star = 0$ in (S1.18), and find

$$\overline{n}^\star = dS_0\varphi\Big(1 + \sum_i \overline{x}_i^{(\tau)}\delta\varphi_i\Big)\ , \qquad \text{(S1.19)}$$

which is Eq. (10) in the main text. We see that the isocline position of the isocline is mostly determined by $dS_0\varphi$. It interpolates between the single strain fixed points on the boundaries, given by Eq. (S1.11). The difference in inoculum size between the two fixed points is $2dS_0\varphi\delta\varphi_1$; thus smaller differences in yield make it actually more unlikely to find a stable fixed point. This can be observed directly in Fig. 4, where the coexistence regions for small $\delta\varphi_1$ becomes a narrow region where $\delta\alpha_1$ and $\delta\varphi_1$ are almost proportional to each other.

The composition isocline is obtained by similar methods. The two terms in its change over cycles, $\Delta\overline{x}_i =$

$\langle \Delta X_i \rangle + \mathbb{C}\text{ov}[X_i, N/\langle N \rangle]$, can be expanded as

$$\langle \Delta X_i \rangle = \left\langle \frac{x_i \xi^{\delta\alpha_i}}{\sum_j x_j \xi^{\delta\alpha_j}} - x_i \right\rangle \tag{S1.20a}$$
$$\approx \delta\alpha_i \langle x_i \log \xi \rangle - \sum_j \delta\alpha_j \langle x_i x_j \log \xi \rangle \ ,$$

$$\mathbb{C}\text{ov}\Big[X_i, \frac{N}{\langle N \rangle}\Big] = \frac{\langle n x_i \xi^{1+\delta\alpha_i} \rangle}{\langle n\xi \big(\sum_j x_j \xi^{\delta\alpha_j}\big) \rangle} - \left\langle \frac{x_i \xi^{\delta\alpha_i}}{\sum_j x_j \xi^{\delta\alpha_j}} \right\rangle$$
$$\approx \langle x_i (W-1) \rangle + \delta\alpha_i \langle x_i (W-1) \log \xi \rangle$$
$$- \sum_j \delta\alpha_j \langle x_i W \rangle \langle x_j W \log \xi \rangle$$
$$+ \sum_j \delta\alpha_j \langle x_i x_j \log \xi \rangle \ , \tag{S1.20b}$$

with $W = n\xi / \langle n\xi \rangle$ denoting relative deviations from expected total sizes. These general expression hold for any interaction between strains encoded in the expansion factor ξ, and we will also use them for the two public good scenarios with antibiotics and pyoverdine.

After inserting ξ into the change of composition surprisingly many terms cancel up to first order of strain differences $\delta\alpha_i$ and $\delta\varphi_i$. The resulting expressions are

$$\langle \Delta X_i \rangle \approx \delta\alpha_i \langle x_i \log \tfrac{S_0 \varphi}{n} \rangle \tag{S1.21a}$$
$$- \sum_j \delta\alpha_j \langle x_i x_j \log \tfrac{S_0 \varphi}{n} \rangle \ ,$$
$$\mathbb{C}\text{ov}[X_i, N/\langle N \rangle] \approx \sum_j \delta\varphi_j \mathbb{C}\text{ov}[x_i, x_j] \ . \tag{S1.21b}$$

For two strains, they simplify to $\langle \Delta X_1 \rangle \approx 2\delta\alpha_1 \langle x_1 (1 - x_1) \log \frac{S_0\varphi}{n} \rangle$ and $\mathbb{C}\text{ov}[X_1, N/\langle N \rangle] \approx 2\delta\varphi_1 (\langle x_1^2 \rangle - \langle x_1 \rangle^2)$. The two independent Poisson distributions for inoculum sizes n_1 and n_2 can be transformed into a Poisson distribution for the total size $n = n_1 + n_2$ and a binomial distribution for n_1 between 0 and n (with parameter $\overline{n}_1/\overline{n}$). Using this, we evaluate the averages

$$\langle x_1^2 \rangle = \sum_{n=1}^{\infty} e^{-\overline{n}} \frac{\overline{n}^n}{n!} \sum_{n_1=0}^{n} \binom{n}{n_1} \overline{x}_1^{n_1} (1-\overline{x}_1)^{n-n_1} \left(\frac{n_1}{n}\right)^2$$
$$= \overline{x}_1^2 + \overline{x}_1 (1 - \overline{x}_1) \langle 1/n \rangle \ ,$$
$$\langle x_1 \rangle = \overline{x}_1 \ ,$$
$$\langle x_1^2 \log n \rangle = \overline{x}_1^2 \langle \log n \rangle + \overline{x}_1 (1-\overline{x}_1) \langle (\log n)/n \rangle \ ,$$
$$\langle x_1 \log n \rangle = \overline{x}_1 \langle \log n \rangle \ .$$

The remaining averages indicated by angular brackets only contain the total population size, while the average over the binomial distribution for $\overline{x}_1$ has been carried out exactly. With these expressions, the composition isocline equation $\Delta \overline{x}_1^\star = 0$ is written as

$$0 \approx \delta\alpha_1 \langle (1 - 1/n) \log \tfrac{S_0\varphi}{n} \rangle + \delta\varphi_1 \langle 1/n \rangle \ ,$$

from which we extract the first order approximation reported in the main text,

$$\overline{n} \approx \frac{|\delta\varphi_1 / \delta\alpha_1|}{\log S_0 \varphi} \ , \tag{S1.22}$$

by neglecting the logarithmic dependence on n, and assuming inoculum sizes are large enough that $\langle 1 - 1/n \rangle \approx 1$ and setting $\langle 1/n \rangle \approx 1/\overline{n}$. Numerical observations suggest that this scaling also contains a coefficient close to 2, due to these neglected terms. However, we are mostly interested in how this position of the composition isocline changes upon variation of the different parameters in the model. A dependence on composition $\overline{x}_1^{(\tau)}$ will only appear in higher orders of $\delta\alpha_1$ and $\delta\varphi_1$, which is consistent with the very straight (orange) lines depicted in Fig. 3 of the main text. We also see that decreasing growth rate differences will push this isocline to larger inoculum sizes. This strong dependence on $\delta\alpha_i$ of the composition isocline is in stark contrast to the position of the population size isocline, which is independent of $\delta\alpha_i$ to first order. The dilution rate d plays no role in the position of the composition isocline, as it cancels already out when calculating the ratio in the cycle mapping, Eq. (S1.6b).

E. Coexistence of multiple strains

This section presents an argument on how multiple strains can coexist in the dynamics. These considerations benefit from a description in absolute numbers $\overline{\mathbf{n}} = (\overline{n}_1, \overline{n}_2, \dots)$ for each of the strains, and not use the non-linear transformation of computing fractions $\overline{x}_i = \overline{n}_i / (\overline{n}_1 + \overline{n}_2 + \dots)$.

One way to define coexistence in our model for multiple strains is when *all* single strain fixed points are unstable with respect to invasion by *every* other strain. This instability of all single strain fixed points implies that the system will have an additional coexistence fixed point. If only a subset of strains fulfill these pairwise condition, such a subset can coexist over cycles. The example for two strains shown in Fig. 3 depicts such cases.

Formally, this occurs when the Jacobian evaluated at these single strain fixed points features eigenvalues larger than 1. Entries of the Jacobian $\mathcal{J}$ at these points $\overline{\mathbf{n}}_i^\star \equiv (0, \dots, \overline{n}_i^\star, \dots, 0)$ can be computed by

$$(\mathcal{J}_{ij})_{\overline{\mathbf{n}}_i^\star} = d\, \partial_{\overline{n}_i} \langle N_j (T_{\text{mix}}; \mathbf{n}) \big| \overline{\mathbf{n}} \rangle \big|_{\overline{\mathbf{n}} = \overline{\mathbf{n}}_i^\star} \ . \tag{S1.23}$$

For the following, we assume that the resident strain has index 1. We already computed the first entry, $\mathcal{J}_{11} = \partial_{\overline{n}_1} \langle N_1(n_1, n_2 \dots) \big| (\overline{n}_1^\star, 0 \dots) \rangle < 1$, in Eq. (S1.12), which just indicates that the resident strain is stable on its own. Other entries in the diagonal indicate an invading strain growing when *each* deme is seeded with exactly one cell of this invading strain,

$$\mathcal{J}_{ii} = d\, \partial_{\overline{n}_i} \langle N_i \big| (\overline{n}_1^\star, 0 \dots) \rangle$$
$$= d \langle N_i (n_1, \dots, 1, \dots) \big| (\overline{n}_1^\star, 0 \dots) \rangle \ , \tag{S1.24}$$

for $i \geq 2$, where we used the auxiliary calculations in Eqs. (S1.9) and (S1.10). Apart from entries on the

diagonal, the Jacobian is a very sparse matrix: Only the first row contains other non-zero entries, which are final population sizes of the resident strain 1 upon invasion of each other strains (with one cell of the invading strain in each deme). Every other entry indicates the change in final population sizes for the other strains: These are all zero and do not change – the Jacobian is evaluated at the single strain fixed point of the resident strain. Thus, the structure of the Jacobian implies that all entries on the diagonal, Eq. (S1.24), are already the eigenvalues. Assuming the system has k strains, each of the k single strain fixed points needs to be unstable with respect to invasion to all $(k-1)$ other strains, leading to $k(k-1)$ (pairwise) conditions of the form $\mathcal{J}_{ii}|_{\overline{n}_j^\star} > 1$.

The coexistence regions depicted in Fig. 4 are obtained by numerically evaluating these conditions for two strains. Then, only the two conditions $\big\langle N_2(n_1,1)\big|\,(\overline{n}_1^\star,0)\big\rangle > 1$ and $\big\langle N_1(1,n_2)\big|\,(0,\overline{n}_2^\star)\big\rangle > 1$ need to be fulfilled.

This set of conditions in Eq. (S1.24) is not constructive: it not indicate how to choose parameters to get coexistence. Only when a set of strains is given by their growth parameters α_i and φ_i, one can check if such a set can coexist. Numerical evaluations indicate the the possible range of values for these two parameters decreases with each additional strain. For example in [1], where a related spatio-temporal model was studied, values were chosen with fine-tuned relations along α and φ to find coexistence between multiple strains.

S1.2. GROWTH DYNAMICS WITH ANTIBIOTICS

In the presence of antibiotics an additional equation for the dynamics of its concentration is coupled to the microbial strain growth and resource consumption dynamics (see main text). This section presents the derivation of the expansion factor ξ for these dynamical interactions through collective antibiotic reduction by extracellular enzymes. With ξ computed, we can use the general expressions for the changes in the inoculum, Eqs. (S1.17) and (S1.20). The additional steps needed to derive the approximations for isoclines reported in the main text are found below.

A. From dynamical interactions within demes to expansion factors

In the presence of antibiotics, the growth rate of strains is given by

$$\alpha_i(t) = \alpha\big(1+\delta\alpha_i\big)\frac{1-(B(t)/\mu)^\kappa}{1+(B(t)/\mu)^\kappa/\gamma}\ . \tag{S1.25}$$

Thus, growth rate is a sigmoidal, decreasing curve for increasing antibiotic concentrations $B(t)$. In this expression, the antibiotic concentration is always measured in multiples of the minimal inhibitory concentration (MIC) μ, which is the concentration where the population switches from overall death to overall growth, such that $\alpha(B=\mu)=0$.

In the within-deme dynamics, the antibiotic concentration $B(t)$ is reduced by the different microbial strains with rates ρ_i. Thus, the set of relevant equations is given by

$$\partial_t N_i = \alpha_i(B) N_i\ , \tag{S1.26a}$$
$$\partial_t B = -\sum_i \rho_i N_i B\ . \tag{S1.26b}$$

For the moment, we ignore the resource dynamics $S(t)$, and ask if the populations in Eq. (S1.26) will survive or go extinct? In a different manuscript, we show that our ensuing approximations are actually very robust against any changes of molecular details, and the results hold for several different dynamics of antibiotic resistance (manuscript in preparation).

In order to solve the combined dynamics of microbial death and antibiotic reduction, the first step involves defining a logarithmic antibiotic concentration $K=\log(B/\mu)$. Often, only the magnitude of the concentration is important, and not its exact value. Using this logarithmic concentration, we can approximate $\alpha_i(B) \approx \frac{\alpha_i\kappa\gamma}{1+\gamma}\log(B/\mu) = -\alpha_i\lambda K$, where we defined the compound parameter $\lambda = \kappa\gamma/(1+\gamma)$. This allows to write the dynamics in Eqs. (S1.26) as $\partial_t N_i = -\alpha_i\lambda K N_i$ and $\partial_t K = -\sum_i \rho_i N_i$. We assume that population sizes decay with a rate determined by the initial antibiotic concentration, $N_i(t) = n_i \exp\big(-\alpha_i\lambda K_0 t\big)$, and this death rate does no change significantly during the relevant time. Numerical integration shows that this approximation is sufficiently close to the actual solution. Thus, with this exponential decay in population size we can integrate the dynamics for K, which yields

$$K(t) = K_0 + \sum_i \frac{n_i\rho_i}{\alpha_i\lambda K_0}\Big(\exp\big(-\alpha_i\lambda K_0 t\big) - 1\Big)\ .$$

Now we have both trajectories, $N(t)$ and $K(t)$, such that we define a time T_1 implicitly via $N(T_1)=1$ and thus indicates when the exponential decay of the population reaches a single cell, $1 = n_i\exp(-\alpha_i\lambda K_0 T_1)$. Solving for t_1 and inserting this expression into the dynamics of the logarithmic antibiotic concentration, $K(T_1)$, we can check if antibiotics have been reduced sufficiently, $K(T_1)<0$ (population survived), or still exceeds this value $K(T_1)>0$ (population will go extinct):

$$0 < K(T_1) = K_0 + \sum_i \frac{n_i\rho_i}{\alpha_i\lambda K_0}\left(\frac{1}{n_i} - 1\right)\ .$$

For large enough inoculum sizes, $1/n_i \ll 1$, we can rearrange this inequality to find

$$0 < \big(\log(B_0/\mu)\big)^2 - n\sum_i \frac{x_i\rho_i}{\alpha_i\lambda}\ . \tag{S1.27}$$

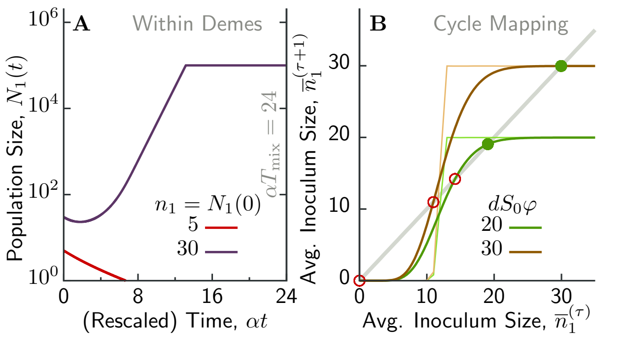

Figure S1.2. **Dynamics of antibiotic reduction for only a single producer strain.** (A) Within demes, the initial population n_1 either manages to reduce antibiotics in time (purple trajectory), or fails and goes extinct (red trajectory). (B) In the mapping of average inoculum sizes over cycles, this threshold in inoculum size between survival and extinction generates a step-like increase of the cycle map. Dashed lines indicate the expected inoculum size if the dynamics within demes would start with an inoculum of n_1, while solid lines indicate the mapping for the average population size $\overline{n}_1$. The latter includes a range of possible inoculum sizes sampled from a Poisson distribution, which smears out the hard cut-off between survival and extinction. Depending on dilution rate d, either two or no (non-zero) fixed points exist. Parameters are $S_0\varphi = 10^5$, $\rho_1/\alpha = 5\cdot 10^{-3}$, $B_0/\mu = 1.25$.

This condition in Eq. (S1.27) indicates that the microbial population reduces the antibiotic concentration fast enough before going extinct. If the population survives, the growing populations it will degrade any remaining antibiotic fast, and $B(t)/\mu$ drops from 1 to 0. We assume that the mixing time T_{mix} is long enough, such that any surviving population will use up all remaining nutrients. The number of dying cells during this initial phase of population death is likely small compared to final population sizes. Thus, these final sizes will be close to the population sizes obtained from the simple resource consumption model, $N \approx S_0\varphi$. Consequently, the expansion factor can be approximated as

$$\xi(T_{\mathrm{mix}}; n, \mathbf{x}) \approx \frac{S_0\varphi}{n}\,\Theta\left[n\sum_i \frac{x_i\rho_i}{\alpha_i\lambda} - \big(\log(B_0/\mu)\big)^2\right]\,. \tag{S1.28}$$

The Heaviside-Theta function Θ in this expression indicates the survival condition (S1.27) above: the expansion factor evaluates to zero, if all cells die, and is given by the first order term $S_0\varphi/n$ of Eq. (S1.16) when they survive.

B. Isoclines of the cycle mapping

In order to fully understand the isoclines for antibiotic reduction in Fig. 5, it helps to investigate the dynamics of single strain first. Fig. S1.2 illustrates this map of inoculum sizes, using a single producing strain. The depicted sigmoidal curve emerges from smearing out the sharp cut-off between surviving and extinct populations in the expansion factor ξ, Eq. (S1.28), due to the Poisson distribution in the seeding step. This shape implies that either one or three fixed points occur in the mapping. Similar to the resource consumption dynamics, extinction is always one of these fixed points, $\overline{n}^\star = 0$. In contrast to before, however, it is usually stable. The other two fixed points are close to the survival threshold, $\overline{n}_1^\star \approx (\alpha_1\lambda/\rho)\big(\log(B_0/\mu)\big)^2$, and the already known fixed point where all resources are used up, $\overline{n}_1^\star \approx dS_0\varphi_1$. The latter fixed point is always stable, if it exists, and the former is always unstable and acts as barrier between the basins of attraction to either extinction or survival, see Fig. S1.2B. These two fixed points only exists, if their expressions assume values such that the survival threshold is smaller than the fixed point where resources are used up. Otherwise, extinction is the only possible long-term outcome in the dynamics.

For two strains, the two single strain fixed points indicate the intersections of the population size isocline with the boundary at $\overline{x}_1 = 1$, see Fig. 5. From the stable fixed point (for resource depletion, with larger inoculum sizes) the isocline extends down to lower $\overline{x}_1$ values along the condition derived before for resource consumption, Eq. (S1.19). Without any differences in yield, $\delta\varphi_1 = 0$, it is a straight line. The other condition, limited by the antibiotic threat, extends along constant producer inoculum sizes, $\overline{n}_1 = const$, and is given by the expression computed above for survival of the population. Thus, this part of the isocline will scale with $\overline{n} \sim \overline{x}_1^{-1}$, where the proportionality factor is determined by the threshold population size. Both parts of the isocline will meet, when $\overline{x}_1$ becomes small enough, and the isoclines connects these two descriptions.

The population composition isocline, $0 = \Delta\overline{x}_1^\star$, is determined by the balance of the local losses in frequency and the coupling to population size changes, $0 = \langle\Delta X_1\rangle + \mathbb{C}\mathrm{ov}\big[X_1, N/\langle N\rangle\big]$. As we assume $\delta\alpha_1 < 0$ for the producing strain, the first term is usually negative. Inspecting the expansion in growth rate differences, see Eq. (S1.20), we observe that only a single term does not depend on $\delta\alpha_1$ – contrary to the resource consumption before, factors of $\delta\alpha_i$ and $\delta\varphi_i$ in ξ are negligible for antibiotic reduction. This term, $\langle x_1(W-1)\rangle$, where $W = n\xi/\langle n\xi\rangle$, is responsible for large values in the covariance, that lead to positive values in the overall change $\Delta\overline{x}_1$, required for coexistence. Inserting ξ for antibiotics the condition for the isocline, $0 = \Delta\overline{x}_1^\star$, can be written as

$$0 = \Big(\langle x_1\Theta\rangle/\langle\Theta\rangle - \langle x_1\rangle\Big) - \mathcal{O}\big(|\delta\alpha_1|\big)\,, \tag{S1.29}$$

where Θ abbreviates the full expression of the threshold in ξ. The first bracket indicates the difference between the average inoculum fraction of the producing strain in surviving populations and its average fraction in the inoculum. Such an expression is expected to be positive,

as surviving populations likely have a larger fraction of the producing strain. However, with increasing inoculum sizes this term will become smaller, as then more populations survive, and the two averages become closer.

When evaluating this first term in Eq. (S1.29) numerically, we find two regimes: For small values of $\overline{n}_1$, it is an exponential decay in $\overline{n}_1$, until $\overline{n}_1$ reaches its threshold value in the argument of Θ. For $\overline{n}_1$ larger than this threshold, the difference decays algebraically with an exponent close to -1. In contrast, the dependence on the non-producer inoculum size $\overline{n}_2$ is weak. Thus, $\overline{n}_1$ already specifies the shape of $\langle x_1(W-1)\rangle$ across the whole phase plane, given by $\langle x_1\Theta\rangle/\langle\Theta\rangle - \langle x_1\rangle \sim 1/\overline{n}_1$ in the relevant region. For the isocline, the decay of this (positive) expression needs to be balanced by all the other terms, which are of order $\mathcal{O}(\delta\alpha_1)$; this coefficient $\delta\alpha_1$ already determines the magnitude, other dependencies only add minor variations to this value. Hence, for the isocline we seek curves where the first term in Eq. (S1.29) is small and almost constant, which occurs along $\overline{n}_1 \approx const$, which is the main dependency of the first term. Thus, we approximate the scaling of the isocline as

$$\Delta\overline{x}^\star = 0 \quad \Leftrightarrow \quad \overline{n} \sim \frac{1}{\delta\alpha_1 \overline{x}_1^{1+\epsilon}}\ , \qquad \text{(S1.30)}$$

which holds as long as enough cells of both strains are in the inoculum. Here, we introduced the small positive constant $\epsilon \ll 1$, which we only observe numerically. It is needed, however, in order to explain that the composition isocline is typically steeper than the inoculum size isocline in phase plane, which also scales as $\sim 1/\overline{x}_1$.

C. Stability of the coexistence fixed point

The two curved isoclines will in general have multiple intersections, which are all fixed points of the cycle mapping. However, we find that at most two of them are stable: A population of only producing strains can survive, as shown for the single strain case above. Such a fixed point appears on the boundary of the phase space, $\overline{x}_1 = 1$.

Coexistence can occur, if the two isoclines intersect in a specific way: on the inoculum size isocline, the intersection has to be on the (vertical) part described by resource limitation. On the composition isocline, the intersection needs to occur on the part following the extinction threshold. Only for an intersection on these parts of the isocline, we find a stable fixed point. In Fig. 6 we showed how these isoclines change when changing either the dilution rate d or the growth rate differences $\delta\alpha_1$. Fig. S1.3 extends these results and shows the stability of this potential coexistence fixed point for a much larger range of parameters. The color encodes the result of a linear stability analysis, obtained by computing eigenvalues of the cycle map evaluated at the fixed point: for constant dilution rate d and increasing the growth rate difference $\delta\alpha_1$ the fixed point goes from stable (green solid), to stable complex (purple solid), to unstable complex (purple circle), and finally to unstable (red circle). Clearly, the actual position in phase plane of these fixed point will change as well, but this information is not displayed in Fig. S1.3. The purple and brown boxes enclose the parameter range shown in more detail already in the main text in Fig. 6. Numerically, we also found stable limit cycles in the unstable complex regime, close to the transition between stable and unstable, from a Neimark-Sacker bifurcation [5]. This transition occurs when the intersection of the two isoclines moves on the inoculum size isocline from the description by resource limitation to the description by antibiotic reduction (at its smallest $\overline{x}_1$ values).

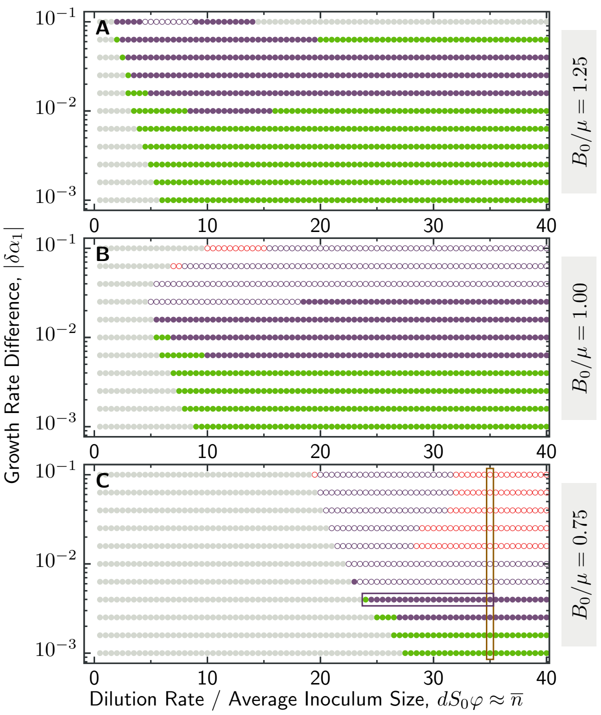

Figure S1.3. **Stability of fixed points within-deme dynamics involving antibiotic hazards.** Displayed are color-coded stability properties of the coexistence fixed point, as long as it exists (gray symbols indicate it does not). Specifically, the colors indicate stable (●), stable with complex eigenvalues (●), unstable with complex eigenvalues (○) and unstable (○) fixed points. In the complex unstable regime, close to the boundary to stability, we also find stable limit cycles, which are not captured in the linear stability analysis of computing eigenvalues of the Jacobian. These limit cycles appear through a Neimark-Sacker bifurcation [5]. The change in shape and position for the two isoclines of inoculum size and inoculum composition are shown in more detail in Fig. 6, where purple and brown boxes in panel (C) correspond to the similar colored borders.

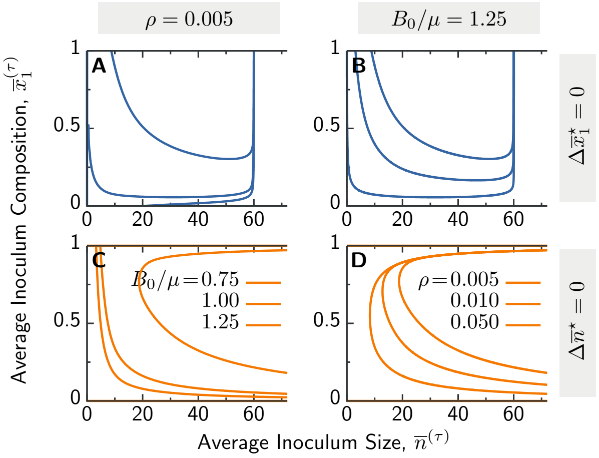

Figure S1.4. **Influence of other parameters on the shape of isoclines in the dynamics with antibiotics.** Panels show the isoclines for (A) average inoculum size $\overline{n}$ and (B) average inoculum composition $\overline{x}_1$, when varying the initial antibiotic concentration B_0/μ. In (A), from top to bottom these initial values are $B_0/\mu = 1.25, 1.00, 0.75$. In (B), the innermost curve indicates the highest antibiotic concentration, while the outermost curve the lowest concentration. Panels (C) and (D) show the effect of changing the production rate ρ_1 of the enzyme. Parameters $dS_0\varphi = 60$ and $\delta\alpha_1 = -10^{-2}$ are constant in all panels.

D. Additional parameter dependence of the cycle mapping

In the main text, we focused on how isoclines change upon varying the two parameters $\delta\alpha_1$ and d. Each of them changes one of the two isoclines (population size or population composition, respectively), while leaving the other isocline almost unaffected. However, the models for antibiotic reduction also features additional parameters, which we explore here.

The dynamics of antibiotic reduction clearly depends on the antibiotic concentration B_0/μ present at the beginning of the growth phase, and how fast antibiotics is reduced. From the threshold between growth/no-growth, as stated in Eq. (S1.27),

$$n \sum_i \frac{x_i \rho_i}{\alpha_i \lambda} > \big(\log(B_0/\mu)\big)^2 , \qquad \text{(S1.31)}$$

we see that both $\rho_i/\alpha_i\lambda$ and B_0/μ give additional important parameter combinations. Fig. S1.4 illustrates the effects of changing either of those. In general, as long as $B_0/\mu > 1$, the effect of changing either production rate or initial antibiotic concentration only changes the threshold inoculum size for producing strains that would survive. Changing this threshold only shifts the position of the isoclines, but does not significantly change their shapes. However, when $B_0/\mu < 1$ the non-producing strain will be able to survive on its own, although it might grow significantly slower in demes where antibiotic concentration is not reduced. Due to this survival of the non-producing strain, the part of the composition isocline that indicated the extinction of the non-producing strain (asymptotic approach towards the $\overline{x}_1 = 1$ axis) disappears, and the composition isocline directly intersects with the pure-producer axis of the phase plane, as shown in Fig. S1.4AB.

S1.3. GROWTH DYNAMICS WITH PYOVERDINE

Similar to the public good dynamics with antibiotics, we compute here an expansion factor ξ that takes the effect of pyoverdine production into account. This allows to use the same approximations employed in previous sections to derive the isoclines. Here, we provide the steps in the derivation of these approximations. In contrast to before, however, we mostly focus on the effect of the public good, and neglect several corrections due to the strain differences in $\delta\alpha_i$ and $\delta\varphi_i$.

A. From dynamical interactions within demes to expansion factors

Pyoverdine changes yield φ on a population level due to enhanced availability of iron. We assumed its effect is given by,

$$\varphi_i(t) = \varphi(1+\delta\varphi_i)\Big(\sigma - (\sigma-1)e^{-P(t)}\Big) , \qquad \text{(S1.32)}$$

where the latter term in brackets is the time-dependent $Y(t)$ in Eq. (S1.2b). The production of pyoverdine P is given by $\partial_t P = \sum_i \rho_i N_i$. In order to compute the effects on the expansion factor ξ, we need to evaluate the full resource-use Eq. (S1.15), which contains an integral over these time-dependent changes in yield, $\int dt S(t)(\partial_t Y)(t)$.

To this end, we combine the two ODEs for $S(t)$ and $P(t)$, which are both linear in $N(t)$. First, we rearrange both to bring $N(t)$ to one side of the respective equation, and then equate both expressions, which leads to

$$\partial_t S = -\underbrace{\left(\alpha \frac{\sum_j \frac{1+\delta\alpha_j}{1+\delta\varphi_j} X_j}{\sum_k \rho_k X_k}\right)}_{\equiv R} \frac{\partial_t P}{\varphi Y(P)} . \qquad \text{(S1.33)}$$

In anticipation of the result we wrote the dynamics of the pyoverdine concentration P together with yield $\varphi Y(P)$, which is influenced by this concentration. The population composition $X_i(t)$ changes only slowly in time, thus we assume that $X_i(t) \approx x_i$ for the purpose of the ensuing approximations, and collect all these dependencies in the term R, which we assume to be roughly constant over time. Then, we can integrate this equation from initial conditions $S(0) = S_0$ and $P(0) = 0$ up to a time t to get the relation between $S(t)$ and $P(t)$,

$$S(t) = S_0 - \frac{R}{\varphi\sigma} \log\Big(1 + \sigma\big(e^{P(t)} - 1\big)\Big) , \qquad \text{(S1.34)}$$

which is an almost linear expression of the form $S(P) \approx S_0 - \frac{R}{\varphi\sigma}P$. As a next step, we take a derivative with respect to time on $\varphi(P)$, Eq. (S1.32), to obtain

$$\partial_t \varphi(t) = \varphi(\sigma-1)(\partial_t P)\exp\big(-P(t)\big)\ . \tag{S1.35}$$

With these two expressions, we transform the integration variable, $dt(\partial_t P) = dP$, together with adjusting the integration bounds, in the full resource-use dynamics, Eq. (S1.15). Then, we can evaluate this equation to obtain

$$\begin{aligned} S_0\varphi + &\int_0^{T_{\rm depl}} dt\ (\partial_t\varphi) S \\ &\approx S_0\varphi + \varphi(\sigma-1)\int_0^{P(T_{\rm depl})} dP\ \Big(S_0 - \frac{R}{\varphi\sigma}P\Big)e^{-P} \\ &= S_0\varphi\Big(\sigma - (\sigma-1)e^{-P(T_{\rm depl})}\Big) \\ &\quad + R\frac{\sigma-1}{\sigma}\Big(1 - e^{-P(T_{\rm depl})}\big(1+P(T_{\rm depl})\big)\Big)\ . \end{aligned}$$

The first line in this solution indicates the expected yield at the end of growth phase. The expression of the second line denotes an correction due to the fact that the concentration of P is increasing during this growth phase, and did not yet start at its final value.

Having this expression, we can simplify further, and neglect the second line to obtain the main scaling of the expansion factor. To this end, we define the abbreviations $\xi_0 = S_0\varphi\sigma/n$ and $H = \sum_i \frac{x_i\xi^{\delta\alpha_i}}{1+\delta\varphi_i}$. The full resource use equation can then be written as

$$\xi_0\left(1 - \frac{\sigma-1}{\sigma}\exp\left(-\frac{n}{R}\xi H\right)\right) = \xi H\ ,$$

which we need to solve for ξ. Note that R also contains a logarithmic dependency on ξ, which does not influence the overall scaling. The term H generates the corrections in $\delta\alpha_i$ and $\delta\varphi_i$, leading to the term G reported in Eq. (S1.16). this generates only multiplicative factors, which is the reason that we solve for ξH first. We obtain

$$\xi H = \xi_0 + \frac{R}{n}\mathscr{W}\left(-\frac{\sigma-1}{\sigma}(n\xi_0/R)e^{-n\xi_0/R}\right)\ .$$

For small enough arguments, the negative branch of the Lambert-W function $\mathscr{W}$ is linear in this argument, which is the case here: $n\xi_0/R$ is usually large. Thus, we can simply leave out $\mathscr{W}$ to obtain a scaling of the expansion factor for pyoverdine production,

$$\xi(T_{\rm depl}; n, \mathbf{x}) \approx \frac{S_0\varphi}{n}\left(\sigma - (\sigma-1)\exp\left(-\frac{S_0\varphi\sigma}{R}\right)\right)\ , \tag{S1.36}$$

which assumes $H \approx 1$. In order to include larger deviations from unity in H, we will obtain terms similar to the corrections G, as in Eq. (S1.16) For the following, we define $A(\mathbf{x}) = S_0\varphi\sigma\sum_i x_i\rho_i/\alpha_i$, which is the full argument in the exponential.

B. Computing the shape of isoclines

The approximation for the expansion factor ξ in Eq. (S1.36) can be inserted in the general expressions for the population size in Eq. (S1.17) and for the population composition in Eq. (S1.20). At first, we observe that saturation of the public goods dynamics occurs as long as $A(\mathbf{x}) \gg 1$. Then, the exponential term in Eq. (S1.36) is small and negligible and we have $\xi \approx S_0\varphi\sigma/n$. Accordingly, the single strain fixed point of a producer strain is $\overline{n}_1^\star \approx dS_0\varphi\sigma$. Since we assume that pyoverdine is not essential for growth, we also find a fixed point for the non-producer strain at $\overline{n}_2^\star \approx dS_0\varphi$. For only two strains, the population size isocline then connects these two single strain fixed points: From the producer fixed point it extends to lower producer fractions along a line given by Eq. (S1.19), with the replacement $\varphi \mapsto \varphi\sigma$ to indicate saturated public good effects. At a fraction given by $A(\overline{x}_1) \approx \mathcal{O}(10^0)$, saturation ceases and final population sizes drop to their base level in absence of pyoverdine. Illustrations in Fig. 7 clearly show this behavior, where the (blue) population size isocline exhibits a sharp bend at small producer fractions.

For the composition isocline, we insert the expression for ξ into Eq. (S1.20). Similar to the case with antibiotics, the main term contributing to a large positive covariance term is $\big\langle x_1(W-1)\big\rangle$, which evaluates to

$$\big\langle x_1(W-1)\big\rangle \approx -\frac{\mathbb{C}\mathrm{ov}\big[x_1, \exp(-A(x_1)\big]}{\frac{\sigma}{\sigma-1} - \big\langle \exp(-A(x_1))\big\rangle}\ . \tag{S1.37}$$

Interestingly, when numerically computing these averages over the Poisson distribution, we find that this expression decays exponentially close to $\exp(-\overline{n}_1)$, when $A(1) \gg 1$. The factor σ only plays a role in the coefficient of this scaling. If $A(1) \ll 1$, the dynamics effectively describes the original resource consumption, as then the pyoverdine production does not influence the dynamics. For intermediate values, $A(1) \approx \mathcal{O}(10^0)$, this expression starts to decay much slower. We focus on the simplest case with a significant effect of the public good, where we can use a similar reasoning as with antibiotic reduction: Eq. (S1.37) needs to be balanced by terms of order $\mathcal{O}(\delta\alpha_1)$, and thus we again seek curves in phase space where this expression is small and almost constant. Thus, with the exponential decay $\sim \exp(-\overline{n}_1)$, such curves are given by $\overline{n}_1 = const$, where the position is strongly determined by the growth rate difference $\delta\alpha_1$. For smaller $\delta\alpha_1$ the condition $0 = \Delta\overline{x}_1$ is met further out the tail of the exponential decay. Approximately, this balance is given by $\exp(-\overline{n}_1) \sim \delta\alpha_1$. Thus, the scaling of the composition isocline can be approximated by

$$\Delta\overline{x}_1^\star = 0 \quad \Leftrightarrow \quad \overline{n} \sim \frac{\log|\delta\alpha_1| + C}{\overline{x}_1}\ , \tag{S1.38}$$

with a logarithmic dependence of $\delta\alpha_1$ on its position, and C the coefficient between terms in the balance.

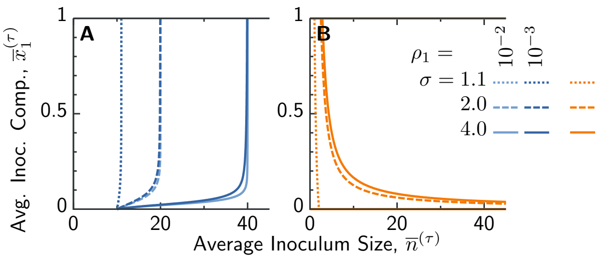

Figure S1.5. **Influence of other parameters on the isoclines in the dynamics with pyoverdine.** Panels show the isoclines for (A) average inoculum size $\overline{n}$ and (B) average inoculum composition $\overline{x}_1$, upon variation of the factor σ that increases yield due to presence of pyoverdine. In panel (A) we additionally illustrate the influence of the production rate ρ_1 as different shades of blue. This production rate does not significantly change the isoclines in (B). Parameters $dS_0\varphi = 10$, $\delta\alpha_1 = -10^{-2}$ are constant for all plots.

C. Other parameter dependence

The main text only explored the effects of changing growth rate differences $\delta\alpha_1$ and the dilution rate d, which are the main parameters for the position of isoclines. Fig. S1.5 explores the effects on isoclines if either of the production rate ρ_1 or the increase in yield σ.

S1.4. NUMERICAL METHODS

Several results in our manuscript have only been obtained numerically, although we tried to explain these results with scaling arguments. Here, we briefly present numerical methods used throughout our work.

In general, the two levels of selection of growth within demes and the mixing cycles are also reflected in our code. We first solve the within-deme dynamics, on a grid of all possible initial conditions (n_1, n_2). These initial conditions are chosen up to values $n_{i,\max}$, such that the probability of finding this combination in the Poisson distribution are tiny and negligible. Often, we use the condition $\mathbb{P}[n_{i,\max}|\overline{n}_i]/\mathbb{P}[\overline{n}_i|\overline{n}_i] \lesssim 10^{-5}$ to determine these maximal inoculum sizes. For most of the results shown in Figs. 5 and 7 this maximal inoculum size is roughly $n_{i,\max} = 200$, which is the value we use in our figures. Solutions to within-deme dynamics are obtained by a standard Runge-Kutta 4th order integration scheme up to the time T_{mix}.

After final population sizes $N_i(T_{\text{mix}}; n_1, n_2)$ are computed, they are stored as lookup-table. Cycles of growth, mixing and reseeding are realized by computing the sums

$$\sum_{n_1,n_2=0}^{n_{i,\max}} \mathbb{P}[n_1|\overline{n}_1]\mathbb{P}[n_2|\overline{n}_2] N_i(T_{\text{mix}}; n_1, n_2) \; .$$

Fixed points of the mapping (e.g. Fig. S1.3) are obtained via iteration of a multi-dimensional Newton-Raphson scheme, which requires usually only a few steps.

Code is available at https://github.com/lukasgeyrhofer/mixingcycles under the CC0 license.

[1] Morten Ernebjerg and Roy Kishony. Dynamic phenotypic clustering in noisy ecosystems. *PLoS computational biology*, 7(3):e1002017, 2011.
[2] Josef Hofbauer and Karl Sigmund. *Evolutionary games and population dynamics.* Cambridge university press, 1998.
[3] James D Murray. *Mathematical Biology. I.* Springer, New York, 1989.
[4] Martin A Nowak. *Evolutionary dynamics.* Harvard University Press, 2006.
[5] Stephen Wiggins. *Introduction to applied nonlinear dynamical systems and chaos.* Springer, 2003.
